# Dynamic Behavior Analysis via Structured Rank Minimization

**DOI:** 10.1007/s11263-016-0985-3

**Published:** 2017-01-19

**Authors:** Christos Georgakis, Yannis Panagakis, Maja Pantic

**Affiliations:** 1 Department of Computing, Imperial College London, 180 Queens Gate, London, SW7 2AZ UK; 2 Department of Computer Science, Middlesex University, London, UK; 3 Faculty of Electrical Engineering, Mathematics and Computer Science (EEMCS), University of Twente, Enschede, The Netherlands

**Keywords:** Dynamic behavior analysis, Structured rank minimization, Linear time-invariant systems, Hankel matrix, Low-rank, Sparsity

## Abstract

Human behavior and affect is inherently a dynamic phenomenon involving temporal evolution of patterns manifested through a multiplicity of non-verbal behavioral cues including facial expressions, body postures and gestures, and vocal outbursts. A natural assumption for human behavior modeling is that a continuous-time characterization of behavior is the output of a linear time-invariant system when behavioral cues act as the input (e.g., continuous rather than discrete annotations of dimensional affect). Here we study the learning of such dynamical system under real-world conditions, namely in the presence of noisy behavioral cues descriptors and possibly unreliable annotations by employing structured rank minimization. To this end, a novel structured rank minimization method and its scalable variant are proposed. The generalizability of the proposed framework is demonstrated by conducting experiments on 3 distinct dynamic behavior analysis tasks, namely (i) conflict intensity prediction, (ii) prediction of valence and arousal, and (iii) tracklet matching. The attained results outperform those achieved by other state-of-the-art methods for these tasks and, hence, evidence the robustness and effectiveness of the proposed approach.

## Introduction

Analysis of human behavior concerns detection, tracking, recognition, and prediction of complex human behaviors including affect and social behaviors such as agreement and conflict escalation/resolution from audio-visual data captured in naturalistic, real-world conditions. Modeling human behavior for automatic analysis in such conditions is the prerequisite for next-generation human-centered computing and novel applications such as personalized natural interfaces (e.g., in autonomous cars), software tools for social skills enhancement including conflict management and negotiation, and assistive technologies (e.g., for independent living), to mention but a few.

Traditionally, research in behavior and affect analysis has focused on recognizing behavioral cues such as smiles, head nods, and laughter (Déniz et al. 2008; Kawato and Ohya 2000; Lockerd and Mueller 2002), pre-defined posed human actions (e.g., walking, running, and hand-clapping) (Dollár et al. 2005; Niebles et al. 2008; Georgakis et al. 2012) or discrete, basic emotional states (e.g., happiness, sadness) (Pantic and Rothkrantz 2000; Cohen et al. 2003; Littlewort et al. 2006) mainly from posed data acquired in laboratory settings. However, these models are deemed unrealistic as they are unable to capture the temporal evolution of non-basic, possibly atypical, behaviors and subtle affective states exhibited by humans in naturalistic settings. In order to accommodate such behaviors and subtle expressions, continuous-time and dimensional descriptions of human behavior and affect have been recently employed (Gunes and Pantic 2010; Gunes et al. 2011; Pantic et al. 2011; Pantic and Vinciarelli 2014; Vrigkas et al. 2015). For instance, the temporal evolution of level of interest (Nicolaou et al. 2014; Panagakis et al. 2016) and agreement (Bousmalis et al. 2011; Rakicevic etal. 2016), or the intensity of pain (Kaltwang et al. 2012, 2015) and conflict (Kim et al. 2012a, b; Panagakis et al. 2016) is precisely described as continuous-valued function of time. In analogy, dimensional and continuous description of human emotion consists of characterizing emotional states in terms of a number of latent dimensions over time (Gunes et al. 2011). Two dimensions are deemed sufficient for capturing most of the affective variability: valence and arousal (V–A), signifying respectively, how positive/negative and active/inactive an emotional state is Lane and Nadel (2002).

Representative machine learning models employed for automatic, continuous behavior and emotion analysis include Hidden Markov Models (HMMs) (Cohen et al. 2003) for facial expression recognition, Dynamic Bayesian Networks (DBN) for human motion classification and tracking (Pavlović et al. 1999), Conditional Random Fields (CRFs) for prediction of visual backchannel cues (i.e., head nods) (Morency et al. 2010), Long-Short Term Memory (LSTM) Neural Networks for continuous prediction of dimensional affect (Nicolaou et al. 2011), and regression-based approaches for continuous emotion and depression recognition or pain estimation (Nicolaou et al. 2012; Valstar et al. 2013; Kaltwang et al. 2012). Despite their merits, these methods rely on large sets of training data, involve learning of a large number of parameters, they do not model dynamics of human behavior and affect in an explicit way, and, more importantly, they are fragile in the presence of gross non-Gaussian noise and incomplete data, which is abundant in real-world (visual) data.

**Fig. 1:**
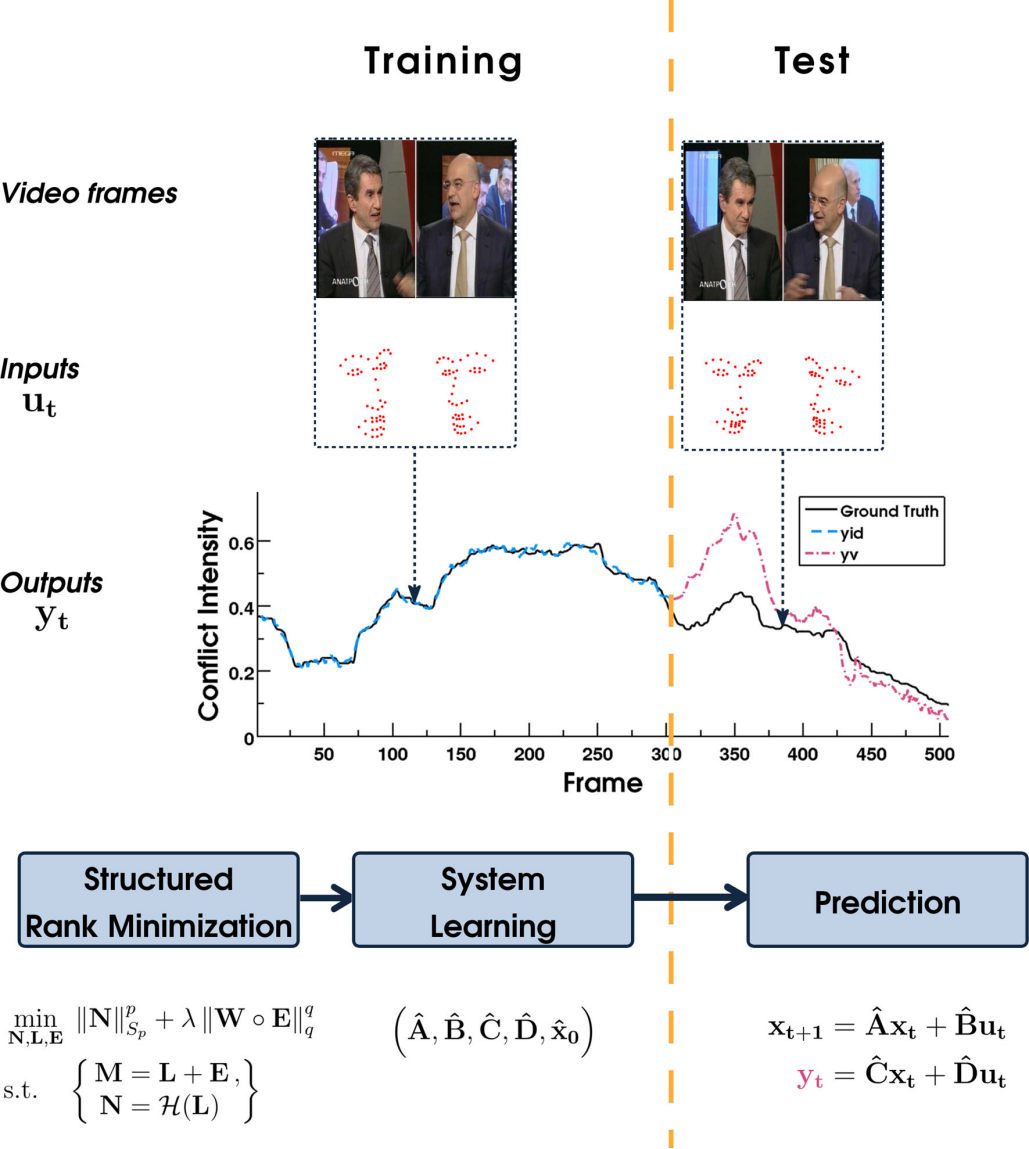
Illustration of the proposed dynamic behavior analysis framework, as applied on the task of conflict intensity prediction for a sequence from CONFER dataset. A portion of the sequence frames is used for LTI system learning through the proposed structured rank minimization method (*training*), while the remaining frames are used for prediction (*test*)

*Contributions* In this work, we model and tackle the problem of *dynamic behavior analysis* in the presence of gross, but sparse noise and incomplete visual data under a different perspective, making the following contributions:

1. The modeling assumption here is that for smoothly-varying dynamic behavior phenomena, such as conflict escalation and resolution, temporal evolution of human affect described in terms of valence and arousal, or motion of human crowds, among others, the observed data can be postulated to be trajectories (inputs and outputs) of a linear time-invariant (LTI) system. Recent advances in system theory (Van Overschee and De Moor 2012; Fazel et al. 2013) indicate that such dynamics can be discovered by learning a low-complexity (i.e., low-order) LTI system based on its inputs and outputs via rank minimization of a Hankel matrix constructed from the observed data. Here, continuous-time annotations characterizing the temporal evolution of relevant behavior or affect are considered as system outputs, while (visual) features describing behavioral cues are deemed system inputs. In practice, visual data are often contaminated by gross, non-Gaussian noise mainly due to pixel corruptions, partial image texture occlusions or feature extraction failure (e.g., incorrect object localization, tracking errors), and human assessments of behavior or affect may be unreliable mainly due to annotator subjectivity or adversarial annotators. The existing structured rank minimization-based methods perform sub-optimally in the presence of gross corruptions. Therefore, to robustly learn a LTI system from grossly corrupted data, we formulate a novel $\ell _q$-*norm regularized (Hankel) structured Schatten-p norm minimization* problem in Sect. 3. The Schatten *p*- and the sparsity promoting $\ell _q$-norm act either as convex surrogates, when $p=q=1$, or as non-convex approximations, when $p,\,q \in (0,1)$, of the rank function and the $\ell _0$-(quasi) norm, respectively.
2. To tackle the proposed optimization problem, an algorithm based on the Alternating-Directions Method of Multipliers (ADMM) (Bertsekas 2014) is developed in Sect. 4. Furthermore, in the same section a scalable version the algorithm is elaborated.
3. The proposed model is the heart of a general and novel framework for dynamic behavior modeling and analysis, which is detailed in Sect. 5. A common practice in behavioral and affective computing is to train machine learning algorithms by employing large sets of training data that comprehensively cover different subjects, contexts, interaction scenarios and recording conditions. The proposed approach allows us to depart from this practice. Specifically, we demonstrate for the first time that complex human behavior and affect, manifested by a single person or group of interactants, can be learned and predicted based on a small amount of person(s)-specific observations, amounting to a duration of just a few seconds.
4. The effectiveness and the generalizability of the proposed model is corroborated by means of experiments on synthetic and real-world data in Sect. 6. In particular, the generalizability of the proposed framework is demonstrated by conducting experiments on 3 distinct dynamic behavior analysis tasks, namely (i) *conflict intensity prediction*, (ii) *prediction of valence and arousal*, and (iii) *tracklet matching*. The attained results outperform those achieved by other state-of-the-art methods on both synthetic and real-world data and, hence, evidence the robustness and effectiveness of the proposed approach. The proposed framework is graphically illustrated in Fig. 1.

## Background and Related Work

In this section, notation conventions and mathematical formalism related to Hankel matrix structure are first introduced. Next, in order to make the paper self-contained, we describe how learning of dynamical systems and, in particular, of a LTI system can be cast as a (Hankel) structured rank minimization problem. Related works on structured rank minimization and their applications in visual information processing are also described.

### Preliminaries

*Notations* Matrices (vectors) are denoted by uppercase (lowercase) boldface letters, e.g., $\mathbf {X, (x)}$. ${\mathbf {I}}$ denotes the identity matrix of compatible dimensions. The *i*th element of vector ${\mathbf {x}}$ is denoted as $x_i$, the *i*th column of matrix ${\mathbf {X}}$ is denoted as $\mathbf {x_i}$, while the entry of $\mathbf {X}$ at position (*i*, *j*) is denoted by $x_{ij}$. For the set of real numbers, the symbol $\mathbb {R}$ is used. For two matrices $\mathbf {A}$ and $\mathbf {B}$ in $\mathbb {R}^{m \times n},$ $\mathbf {A} \circ \mathbf {B}$ denotes the Hadamard (entry-wise) product of $\mathbf {A}$ and $\mathbf {B}$, while $\langle \mathbf {A}, \mathbf {B} \rangle$ denotes the inner product $\text {tr}(\mathbf {A}^T\mathbf {B})$, where $\text {tr}(\cdot )$ is the trace of a square matrix. For a symmetric positive semi-definite matrix $\mathbf {A}$, we write $\mathbf {A} \succeq 0$. Regarding vector norms, $\Vert {\mathbf {x}}\Vert := \sqrt{\sum _{i}{x^2_i}}$ denotes the Euclidean norm. The sign function is denoted by $\text {sgn}(\cdot )$, while $\vert \cdot \vert$ denotes the absolute value operator. Regarding matrix norms, the $\ell _0$- (quasi-) norm, which equals the number of non-zero entries, is denoted by $\Vert \cdot \Vert _0$. $\Vert {\mathbf {X}}\Vert _{q}:= \left( \sum _i \sum _j \vert X_{ij} \vert ^q \right) ^{1/q}$ is the matrix $\ell _q$-norm, of which the Frobenius norm $\Vert {\mathbf {X}}\Vert _{F} := \sqrt{\sum _{i} \sum _{j} X^2_{ij}} = \sqrt{\text {tr}({\mathbf {X}}^{T}{\mathbf {X}})}$ is a special case when $q=2$. $\Vert {\mathbf {X}}\Vert$ denotes the spectral norm, which equals the largest singular value. If $\sigma _i(\mathbf {X})$ is the *i*th singular value of $\mathbf {X}$, $\Vert \mathbf {X}\Vert _{S_p} := \left( \sum _{i}{\sigma _i(\mathbf {X})^p}\right) ^{1/p}$ is the Schatten *p*-norm of $\mathbf {X}$, of which the nuclear norm $\Vert {\mathbf {X}}\Vert _{*} :=\sum _{i}{\sigma _i(\mathbf {X})}$ is a special case when $p=1$. Linear maps are denoted by scripted letters. For a linear map $\mathcal {A}: \mathbb {R}^{m \times n} \rightarrow \mathbb {R}^{p}$, $\mathcal {A}^{*}$ denotes the adjoint map of $\mathcal {A}$, while $\sigma _{\text {max}}(\mathcal {A})$ denotes the maximum singular value of $\mathcal {A}$. $\mathcal {I}$ denotes the identity map.

*The Hankel Matrix Structure* Let $\mathbf {A} = [\mathbf {A_0} \; \mathbf {A_1} \; \ldots \; \mathbf {A_{j+k-2}}]$ be a $m \times n(j+k-1)$ matrix, with each $\mathbf {A_t}$ being a $m \times n$ matrix for $t=0, 1, \ldots , j+k-2$. We define the Hankel linear map ${\mathcal {H}}({\mathbf {A}}):= H_{m,n,j,k}({\mathbf {A}})\varvec{\Gamma }$, where

1 $$\begin{aligned} H_{m,n,j,k}({\mathbf {A}}) = \begin{pmatrix} \mathbf {A_{0}} &{} \mathbf {A_{1}} &{} \cdots &{} \mathbf {A_{k-1}}\\ \mathbf {A_{1}} &{} \mathbf {A_{2}} &{} \cdots &{} \mathbf {A_{k}} \\ \vdots &{} \vdots &{} \ddots &{} \vdots \\ \mathbf {A_{j-1}} &{} \mathbf {A_{j}} &{} \cdots &{} \mathbf {A_{j+k-2}} \end{pmatrix} \in \mathbb {R}^{mj \times nk}, \end{aligned}$$

and $\varvec{\Gamma } \in \mathbb {R}^{n k \times q}$ with $\sigma _{\text {max}}(\varvec{\Gamma }) \le 1$ (Fazel et al. 2013). Therefore, $H_{m,n,j,k}({\mathbf {A}})$ is a block-Hankel matrix with $j \times k$ blocks, where each $\mathbf {A_i}$ is a matrix of dimension $m \times n$. Note that the Hankel structure enforces constant entries along the skew diagonals. We denote by $T = j+k-1$ the total number of observations, while $M = mj$ and $N = nk$ denote the number of rows and columns of the Hankel matrix $H_{m,n,j,k}({\mathbf {A}})$, respectively. For notational convenience, we write $H({\mathbf {A}})$ to denote $H_{m,n,j,k}({\mathbf {A}})$, when the dimensions *m*, *n*, *j*, *k* are clear from the context.

The adjoint map ${\mathcal {H}}^{*}$ is defined as ${\mathcal {H}}^{*}(\varvec{\Lambda }){=} H^{*}_{m,n,j,k}({\varvec{\Lambda }\varvec{\Gamma }^T})$,

where for any matrix ${\mathbf {B}} \in \mathbb {R}^{mj \times nk}$

2 $$\begin{aligned}&H^{*}_{m,n,j,k}({\mathbf {B}}) = H^{*}_{m,n,j,k} \begin{pmatrix} \mathbf {B_{00}} &{} \mathbf {B_{01}} &{} \cdots &{} \mathbf {B_{0,k-1}} \\ \mathbf {B_{10}} &{} \mathbf {B_{11}} &{} \cdots &{} \mathbf {B_{1,k-1}} \\ \vdots &{} \vdots &{} \ddots &{} \vdots \\ \mathbf {B_{j-1,0}} &{} \mathbf {B_{j-1,1}} &{} \cdots &{} \mathbf {B_{j-1,k-1}} \end{pmatrix} \nonumber \\&\quad = \big [\mathbf {B_{00}} \quad \mathbf {B_{01}}+\mathbf {B_{10}} \ldots \nonumber \\&\mathbf {B_{02}}+\mathbf {B_{11}}+\mathbf {B_{20}} \quad \cdots \quad \mathbf {B_{j-1,k-1}} \big ] \in \mathbb {R}^{m \times n(j+k-1)}. \end{aligned}$$

It is proved in Fazel et al. (2013) that $\left||H^{*}_{m,n,j,k}({\mathbf {B}}) \right||^2_F \le L \left|| {\mathbf {B}} \right||^2_F$, where $L:= \text {min}\{j,k\}$. This finding, combined with $\sigma _{\text {max}}(\varvec{\Gamma }) \le 1$, entails that the spectral norm of the adjoint map $\mathcal {H}^{*}$ is less than or equal to $\sqrt{L}$. Herein, the space of Hankel matrices is denoted by $\mathbb {S}_{{\mathcal {H}}}$.

### LTI System Learning via Structured Rank Minimization

Dynamical systems, such as LTI systems, are able to compactly model the temporal evolution of time-varying data. While the dynamic model can be considered as known in some applications (e.g., Brownian dynamics in motion models), it is in general unknown and, hence, should be learned from the available data.

Consider a sequence of observed outputs $\mathbf {{y}_t} \in \mathbb {R}^{m}$ and inputs $\mathbf {{u}_t} \in \mathbb {R}^{d}$, respectively, for $t=0,\ldots ,T-1$. The goal is to find from the observed data, a state-space model, corresponding to a LTI system, given by

3 $$\begin{aligned} \begin{aligned}&\mathbf {x_{t+1}} = {\mathbf {A}}\mathbf {x_t} + {\mathbf {B}}\mathbf {u_t} \\&\mathbf {y_{t}} = {\mathbf {C}}\mathbf {x_t} + {\mathbf {D}}\mathbf {u_t} \, \end{aligned} \end{aligned}$$

such that the system is of low-order, i.e., it is associated with a low-dimensional state vector $\mathbf {x_t} \in \mathbb {R}^{n}$ at time *t*, where *n* is the *unknown* true system order. The order of the system (i.e., the dimension of the state vector) captures the memory of the system and it is a measure of its complexity. In (3), both the state and the measurement equations are linear and the parameters of the system, i.e., the matrices $\mathbf {A,B,C,D}$ are constant over time but their dimensions are *unknown*. Therefore, to determine the model, we need to find the model order *n*, the matrices $\mathbf {A, B, C, D}$, and the initial state $\mathbf {x_0}$. To this end, the model order should be estimated first. Next, the estimation of the system order using Hankel matrices is summarized.

Let us assume that the unknown state vectors has dimension $r > n$ and let $\displaystyle \mathbf {X} = [\mathbf {x_0} \quad \mathbf {x_1} \quad \ldots \quad \mathbf {x_{T-1}}] \in \mathbb {R}^{r \times T}$, $\mathbf {Y} = [\mathbf {y_0} \quad \mathbf {y_1} \quad \ldots \quad \mathbf {y_{T-1}}] \in \mathbb {R}^{m \times T}$, $\mathbf {U} = [\mathbf {u_0} \quad \mathbf {u_1} \quad \ldots \quad \mathbf {u_{T-1}}] \in \mathbb {R}^{d \times T}$ be the matrices containing in their columns the unknown state vectors, the observed outputs, and the observed inputs of the system, respectively, for $t=0,1,\dots ,T-1$. Let also $H_{m,1,r+1,T-r}(\mathbf {Y})$ and $H_{d,1,r+1,T-r}(\mathbf {U})$ be the Hankel matrices constructed from the observed system outputs and inputs, respectively, according to (1) and $\mathbf {U^\perp } \in \mathbb {R}^{(T-r) \times q}$ be the matrix whose columns form an orthogonal basis for the nullspace of $H_{d,1,r+1,T-r}(\mathbf {U})$. Then, the LTI in (3) can be expressed by employing the above mentioned Hankel matrices as follows.

4 $$\begin{aligned} H_{m,1,r+1,T-r}(\mathbf {Y}) = {\mathbf {G}}{\mathbf {X}}+\mathbf {L}H_{d,1,r+1,T-r}(\mathbf {U}) , \end{aligned}$$

where

5 $$\begin{aligned} {\mathbf {G}} = \begin{pmatrix} \mathbf {C} \\ \mathbf {C}\mathbf {A} \\ \vdots \\ \mathbf {C}\mathbf {A^r} \end{pmatrix},&\mathbf {L} = \begin{pmatrix} \mathbf {D} &{} \mathbf {0} &{} \mathbf {0} &{} \cdots &{} \mathbf {0} \\ \mathbf {CB} &{} \mathbf {D} &{} \mathbf {0} &{} \cdots &{} \mathbf {0} \\ \mathbf {CAB} &{} \mathbf {CB} &{} \mathbf {D} &{} \cdots &{} \mathbf {0} \\ \vdots &{} \vdots &{} \vdots &{} \ddots &{} \vdots \\ \mathbf {CA^{r-1}B} &{} \mathbf {CA^{r-2}B} &{} \cdots &{} \cdots &{} \mathbf {D} \end{pmatrix} \end{aligned}$$

By right-multiplying both sides of (4) with $\mathbf {U^\perp }$ and by setting $\mathcal {H}(\mathbf {Y}) = H(\mathbf {Y})\mathbf {U^\perp }$ we obtain

6 $$\begin{aligned} \mathcal {H}(\mathbf {Y}) = {\mathbf {G}}{\mathbf {X}}\mathbf {U^\perp }. \end{aligned}$$

If the inputs are persistently exciting (i.e., ${\mathbf {X}}\mathbf {U^\perp }$ has full rank) and the outputs are exact, then by (6) it is clear that the system order, which is measured by the rank of $\mathbf {G}$ (Van Overschee and De Moor 2012), is equal to $rank \left( \mathcal {H}(\mathbf {Y}) \right)$ (Van Overschee and De Moor 2012) and from it a system realization (i.e., estimation of the unknown system parameters) is easily computed by solving a series of systems of linear equations following, for example, Van Overschee and De Moor (2012).

However, real-world data are not exact and thus $\mathcal {H}(\mathbf {Y})$ is full-rank. Therefore, to find the minimum order realization of the system, we seek a matrix $\hat{\mathbf {Y}}$ which is as close as possible, in the least square sense, to the observed data and the rank of $\mathcal {H}({\hat{\mathbf{Y}}})$ is minimal. Formally, we seek to solve the following Hankel structured rank minimization problem

7 $$\begin{aligned} \min _{\hat{\mathbf {Y}}}{\ \text {rank}(\mathcal {H}({\hat{\mathbf{Y}}})) + \frac{\lambda }{2} \Vert {\hat{\mathbf {Y}}} - \mathbf {Y}} \Vert _{F}^2, \end{aligned}$$

where $\lambda >0$. Assuming that ${\hat{\mathbf {Y}}}$ is a solution of (7), then $\text {rank}(\mathcal {H}({\hat{\mathbf{Y}}}))$ acts as the estimated system order^1^ and ${\hat{\mathbf {Y}}}$ is used next to estimate the system parameters ${\hat{\mathbf {A}},\hat{\mathbf {B}},\hat{\mathbf {C}},\hat{\mathbf {D}}}$ and the initial state vector ${\hat{\mathbf {x}}_0}$ by solving a series of systems of linear equations (Van Overschee and De Moor 2012).

### Hankel Rank Minimization Models and Applications

Problem (7) is combinatorial due to the discrete nature of the rank function and thus difficult to be solved (Fazel et al. 2001). To tackle this problem, several approximations have been proposed. In particular, by employing the nuclear norm, which is the convex surrogate of the rank function (Fazel et al. 2001), a convex approximation of (7) has been proposed in Fazel et al. (2013). By adopting the variational norm of the nuclear norm (i.e., $\Vert {\hat{\mathbf {Y}}} \Vert _* = \min _{{\hat{\mathbf {Y}}} = \mathbf {U}\mathbf {V}} \Vert \mathbf {U} \Vert _F^2 + \Vert \mathbf {V} \Vert _F^2$), non-linear approximations to (7) have been developed (Signoretto et al. 2013; Yu et al. 2014). Furthermore, to estimate the rank of an incomplete Hankel matrix (i.e., in the presence of missing data), the models in Markovsky (2014), Dicle et al. (2013) and Ayazoglu et al. (2012) have been proposed. Representative structured rank minimization models along with the optimization problems that they solve are listed in Table 1.

The aforementioned models have been mainly applied in the fields of system analysis and control theory for *system identification and realization* and in finance for *time-series analysis and forecasting*. More recently, learning dynamical models via Hankel rank minimization has been exploited to address computer vision problems such as *activity recognition* (Li et al. 2011; Bhattacharya et al. 2014), *tracklet matching* (Ding et al. 2007a, 2008; Dicle et al. 2013), *multi-camera tracking* (Ayazoglu et al. 2011), *video inpainting* (Ding et al. 2007b), *causality detection* (Ayazoglu et al. 2013), and *anomaly detection* (Surana et al. 2013). However, none of these methods has been exploited to learn behavior dynamics based on continuous annotations of behavior or affect and visual features. This will be investigated shortly in Sect. 6.

*Remark* Despite their merits, the aforementioned models exhibit the following limitations. By adopting the least squares error, the majority of the models in Table 1 assume Gaussian distributions with small variance (Huber 2011). Such an assumption rarely holds in real-world data that are often corrupted by sparse, non-Gaussian noise (cf. Sect. 1). This drawback is partially alleviated in SRPCA (Ayazoglu et al. 2012), where a sparsity promoting norm is incorporated into the nuclear norm minimization problem in order to account for sparse noise of large magnitude. Furthermore, the convex relaxation of the rank function with the nuclear norm in Fazel et al. (2013) and Ayazoglu et al. (2012) may introduce a relaxation gap. Therefore, due to the above reasons, the estimated rank of the Hankel matrix obtained by the models in Fazel et al. (2013) and Ayazoglu et al. (2012) may be arbitrarily away from the true one (Dai and Li 2014). On the other hand, since the models in Signoretto et al. (2013), Yu et al. (2014) and Markovsky (2014) rely on factorizations of the Hankel matrix, they implicitly assume that the rank of the Hankel matrix is known in advance; obviously this is not the case in practice. To alleviate the aforementioned limitations and robustly estimate the rank of the Hankel matrix in the presence of gross noise and missing data, a novel structured rank minimization model is detailed next.

**Table 1:** List of structured rank minimization methods (including the proposed method) and the corresponding optimization problems

Method	Optimization problem	Convex	Robust
Approximations of (7)			
Proposed	$\min _{\mathbf {L,E}}{\ \left||\mathcal {H}(\mathbf {L}) \right||^{p}_{S_p} + \lambda \left||\mathbf {W} \circ \mathbf {E} \right||^{q}_{q}} \quad \text {s.t.} \quad \mathbf {M} = \mathbf {L}+\mathbf {E} \,$.	depends on the choice of *p* and *q*	✔
Hankel Rank Minimization (HRM) (Fazel et al. 2013)	$\min _{\mathbf {L}}{\frac{1}{2}\left||\mathbf {M} - {\mathcal {A}}(\mathbf {L}) \right||^2_F} + \lambda \Vert \mathcal {H}(\mathbf {L}) \Vert _{*},$ where ${\mathcal {A}}$ is a linear map.	✔	✗
SVD-free (Signoretto et al. 2013)	$\min _{{\mathbf {L}},{\mathbf {Q}},{\mathbf {R}}}{\frac{1}{2}(\mathbf {M}-\mathbf {L})^{T}\mathbf {W}(\mathbf {M}-\mathbf {L}) + \frac{1}{2}(\left||\mathbf {Q} \right||^2_F +\left||\mathbf {R} \right||^2_F )} \quad \text {s.t.} \quad \mathcal {H}(\mathbf {L}) = \mathbf {Q}\mathbf {R}^T.$	✗	✗
Yu et al. (2014)	$\min _{\mathbf {Q},\mathbf {R}}\frac{1}{2}(\left||\mathcal {A}(\mathbf {C}\mathbf {g})-\mathbf {J} \right||^2_F + \frac{\lambda }{2}(\left||\mathbf {J}\mathbf {g} \right||^2_F+ \frac{\mu }{2}(\left||\mathbf {Q} \right||^2_F +\left||\mathbf {R} \right||^2_F ),$	✗	✗
	$$\text {where }\mathbf {g}=\mathbf vec (\mathbf {Q}\mathbf {R}^T)\text { and } {\mathcal {A}} \text { is a linear map.} \text { Return }{\hat{\mathbf {H}}} = \mathbf {Q}\mathbf {R}^T$$.		
Structured Robust PCA (SRPCA) (Ayazoglu et al. 2012)	$\min _{\hat{\mathbf {H}},{\mathbf {E}}}{\sum _i{w_i\sigma _i(\hat{\mathbf {H}})}+\Vert \mathbf {W_e} \circ {\mathbf {E}} \Vert _1 + \frac{1}{2}\Vert \mathbf {W_F} \circ {\mathbf {E}} \Vert ^2_F }$	✔	✔
	$$\text {s.t.} \quad {\mathbf {H}} = {\hat{\mathbf {H}}} + {\mathbf {E}} \;\; \text {;} \;\; \hat{\mathbf {H}}, \, {\mathbf {E}} \in \mathbb {S}_{{\mathcal {H}}}$$.		
Related methods			
Iterative Hankel Total Least Squares (IHTLS) (Dicle et al. 2013)	Given $\mathbf {H} = [\mathbf {F} \, | \, \mathbf {g}] \in \mathbb {S}_{{\mathcal {H}}}$, estimate ${\hat{\mathbf {H}}} = [\mathbf {F}+\mathbf {E} \, | \, \mathbf {g} +\mathbf {k}]$ by solving	✗	✗
	$$\min _{\mathbf {x},\mathbf {E},\mathbf {k}}{\left||\mathbf {W} \circ [\mathbf {E} \, | \, \mathbf {k}] \right||^2_F}$$		
	$$\text {s.t.} \quad (\mathbf {F}+\mathbf {E})\mathbf {x} = \mathbf {g}+\mathbf {k} \;\; \text {;} \;\; [\mathbf {F} \, | \, \mathbf {g}], \, [\mathbf {E} \, | \, \mathbf {k}] \in \mathbb {S}_{{\mathcal {H}}}$$.		
Structured Low-Rank Approximation (SLRA) (Markovsky 2014)	$\min _{\mathbf {G} } F({\mathbf {G}}) \quad \text {s.t.} \quad \mathbf {G} \in \mathbb {R}^{(M-K) \times M} \text { has full row rank, }$	✗	✗
	$$\text {where } F({\mathbf {G}}) := \min _{\mathbf {L}}{\left||\mathbf {W} \circ (\mathbf {M}-\mathbf {L}) \right||^2_F} \quad \text {s.t.} \quad {\mathbf {G}}\mathcal {H}(\mathbf {M})={\mathbf {0}}$$.		

For all methods, the observed data matrix, its Hankel version, and the estimated (Hankel) structured low-rank approximate are denoted by $\mathbf {M} \in \mathbb {R}^{D \times T}$, $\mathbf {H} = \mathcal {H}(\mathbf {M}) \in \mathbb {R}^{M \times N}$ and ${\hat{\mathbf {H}}}=\mathcal {H}(\mathbf {L}) \in \mathbb {R}^{M \times N}$, respectively, unless otherwise stated

## Problem Formulation

Let $\mathbf {M} = [\mathbf {m_0} \; \mathbf {m_1} \; \ldots \; \mathbf {m_{T-1}}] \in \mathbb {R}^{D \times T}$ be a matrix containing in its columns contaminated by gross but sparse noise, time varying data. The goal is to robustly learn the dynamics underlying the data, in the presence of sparse, non-Gaussian noise and missing data.

To this end, we seek to decompose $\mathbf {M}$ as a superposition of two matrices: $\mathbf {M} = \mathbf {L} + \mathbf {E}$, where $\mathbf {L} \in \mathbb {R}^{D \times T}$ and $\mathbf {E} \in \mathbb {R}^{D \times T}$, such that the Hankel matrix of $\mathbf {L}$ (i.e., $\mathcal {H}(\mathbf {L}) \in \mathbb {R}^{M \times N}$) be of minimum rank and $\mathbf {E}$ be sparse. The minimum rank of $\mathcal {H}(\mathbf {L})$ correspond to the minimum-order LTI system that describes the data, while by imposing $\mathbf {E}$ to be sparse, we account for sparse, non-Gaussian noise.

A natural estimator accounting for the low-rank of the Hankel matrix $\mathcal {H}(\mathbf {L})$ and the sparsity of $\mathbf {E}$ is to minimize the rank of $\mathcal {H}(\mathbf {L})$ and the number of non-zero entries of $\mathbf {E}$, measured by the $\ell _0$ (quasi)-norm. This is equivalent to solving the following non-convex optimization problem.

8 $$\begin{aligned} \min _{\mathbf {L}}{\ \text {rank}(\mathcal {H}(\mathbf {L})) + \lambda \Vert \mathbf {M} - \mathbf {L}\Vert _{0}}, \end{aligned}$$

where $\lambda$ is a positive parameter. Clearly, (8) is a robust version of the Hankel structured rank minimization problem (7).

Problem (8) is intractable, as both rank and $\ell _0$-norm minimization are NP-hard (Vandenberghe and Boyd 1996; Natarajan 1995). In order to tackle this NP-hard problem, both convex and non-convex relaxations of the rank function and the $\ell _0$-norm are considered. To this end, we choose to approximate the rank function and the $\ell _0$-norm by the Schatten *p*- and the $\ell _q$-norm, respectively, and solve

9 $$\begin{aligned} \min _{\mathbf {L}}{\ \left||\mathcal {H}(\mathbf {L}) \right||^{p}_{S_p} + \lambda \left||\mathbf {M} - \mathbf {L} \right||^{q}_{q}}, \end{aligned}$$

which is a convex optimization problem for $p=q=1$ (i.e., the Schatten 1-norm is by definition the nuclear norm) and non-convex for $0<p,\,q<1$.

Convex approximations of the rank function and the $\ell _0$-(quasi)-norm by means of the nuclear norm (i.e., Schatten 1-norm) (Fazel et al. 2001) and the $\ell _1$-norm (Donoho 2006) have been widely applied in several rank and sparsity minimization problems (e.g., Candès et al. 2011). The main advantage of this approach is that the global optimum of the convex problems can be found relatively easily by using off-the-shelf optimization methods such as the ADMM. However, the convexification of rank minimization problems may suffer from the following two drawbacks. First, the recoverability of the low-rank solutions via nuclear norm minimization is only guaranteed under *incoherence assumptions* (e.g., Candès et al. 2011). Such assumptions regarding incoherence may not be guaranteed in practical scenarios (Dai and Li 2014). For example in the proposed model, the resulting global optimal solution of the convex instance of (9) ($p,\,q \ge 1$) may be arbitrarily away from the actual solution of (8). Second, it is known that the $\ell _1$-norm is a biased estimator (e.g., Zhang 2010). Since the nuclear norm (or equivalently the Schatten-1 norm) is essentially the application of the $\ell _1$ norm on the singular values, it may only find a biased solution. To alleviate the aforementioned issues of the convex instance of (9), we further consider the non-convex approximation of (8) by employing the Schatten-*p* norm and $\ell _q$-norm with $p,\,q \in (0,1)$. Such non-convex functions have been shown to provide better estimation accuracy and variable selection consistency (Wang et al. 2014b) in related approximations of $\ell _0$-norm regularized rank minimization problems (Nie et al. 2012, 2013; Papamakarios et al. 2014).

To disentangle the Schatten *p*- and $\ell _q$-norm minimization sub-problems in (9) from the matrix structure and data-fitting requirements, respectively, (9) is equivalently written as

10 $$\begin{aligned} \min _{\mathbf {N,L,E}}{\ \left||\mathbf {N} \right||^{p}_{S_p} + \lambda \left||\mathbf {E} \right||^{q}_{q}} \quad \text {s.t.} \quad \begin{Bmatrix} \begin{aligned} \mathbf {M}&= \mathbf {L}+\mathbf {E} \\ \mathbf {N}&= \mathcal {H}(\mathbf {L}) \end{aligned} \end{Bmatrix}. \end{aligned}$$

To account also for (partially) missing observations in $\mathbf {M}$, we introduce the matrix $\mathbf {W} \in \mathbb {R}^{D \times T}$ which is given by

11 $$\begin{aligned} w_{ij} = {\left\{ \begin{array}{ll} 1, &{}\quad \text {if} \ (i,j) \in \Omega , \\ 0, &{}\quad \text {otherwise}, \end{array}\right. } \end{aligned}$$

where $\Omega \subset [1,D] \times [1,T]$ is the set containing the indices of the observed (available) entries in $\mathbf {M}$. By incorporating $\mathbf {W}$ inside the $\ell _q$-norm term in (10) as a multiplicative weight matrix for $\mathbf {E}$, we arrive at the following problem.

12 $$\begin{aligned} \min _{\mathbf {N,L,E}}{\ \left||\mathbf {N} \right||^{p}_{S_p} + \lambda \left||\mathbf {W} \circ \mathbf {E} \right||^{q}_{q}} \quad \text {s.t.} \quad \begin{Bmatrix} \begin{aligned} \mathbf {M}&= \mathbf {L}+\mathbf {E} \\ \mathbf {N}&= \mathcal {H}(\mathbf {L}) \end{aligned} \end{Bmatrix}. \end{aligned}$$

*Remark* Note that the choice of the Hankel map $\mathcal {H}(\cdot )$ depends on the application (see Sects. 2.2, 5). In any case, the Hankel matrix $H_{D,1,j,k}(\mathbf {L}) \in \mathbb {R}^{(M=Dj) \times (N=k)}$ is computed according to (1); the number of blocks along the row and column dimension *j* and *k*, respectively, are set to $j=r+1$ and $T-r$, where *T* is the number of observations and $r > n$, with *n* denoting the system order.

## Algorithmic Frameworks

In this section, the proposed Alternating-Directions Method of Multipliers (ADMM)-based (Bertsekas 2014) solver is described along its scalable version.

### Alternating-Direction Method-Based Algorithm

The ADMM is employed to solve (12). To this end, the augmented Lagrangian function for (12) is defined as follows.

13 $$\begin{aligned} \begin{aligned} \displaystyle&{\mathcal {L}}({\mathbb {V},\mathbb {Y},\mu }) \, = \left||{\mathbf {N}} \right||^{p}_{S_p}+\lambda \left||{\mathbf {W}} \circ {\mathbf {E}} \right||^{q}_{q} \\&+\left\langle {\mathbf {M} - \mathbf {L}-\mathbf {E},{\varvec{\Lambda }_1}}\right\rangle +\left\langle {\mathbf {N} - \mathcal {H}(\mathbf {L}),{\varvec{\Lambda }_2}}\right\rangle \\&+ \frac{\mu }{2}\Big ( \left|| \mathbf {M} - \mathbf {L}-\mathbf {E} \right||^2_F +\left|| \mathbf {N} - \mathcal {H}(\mathbf {L}) \right||^2_F \Big ), \end{aligned} \end{aligned}$$

where $\mu$ is a positive parameter and $\mathbb {V}:=\{{\mathbf {N}} \in \mathbb {R}^{M \times N},{\mathbf {L}} \in \mathbb {R}^{D \times T},{\mathbf {E}} \in \mathbb {R}^{D \times T}\}$, $\mathbb {Y}:=\{{\varvec{\Lambda }_1} \in \mathbb {R}^{D \times T},{\varvec{\Lambda }_2} \in \mathbb {R}^{M \times N}\}$ are the sets containing all the unknown variables and the Lagrange multipliers for the equality constraints in (12), respectively. Specifically, at each iteration of the proposed ADMM-based solver, (13) is minimized with respect to each variable in $\mathbb {V}$ in an alternating fashion and, subsequently, the Lagrange multipliers in $\mathbb {Y}$ and the parameter $\mu$ are updated. The iteration index is denoted herein by *i*. The notation $\mathbb {L}(\mathbf {N} ,\mathbb {Y}[i],\mu [i])$ is used to denote the solution stage in which all other variables but $\mathbf {N}$ are kept fixed, and similarly for the other unknown variables.

The solutions of minimization of (13) with respect to $\mathbf {E}$ and $\mathbf {N}$ are based on the operators and Lemmas that are introduced next. Minimizing (13) with respect to $\mathbf {L}$ does not admit a closed form solution due to the presence of the quadratic terms. Similarly to Fazel et al. (2013), to ‘cancel out’ these terms we add a proximal term to the respective partial augmented Lagrangian. The additive term is based on the (semi-) norm $\left||\cdot \right||_{\mathcal {Q}_0}$ induced by the (semi-) inner product $\mathbf {P}^T\mathcal {Q}_0\mathbf {P}$, with $\mathcal {Q}_0$ being the positive (semi-) definite matrix given by

14 $$\begin{aligned} \mathcal {Q}_0=L\mathcal {I} - {\mathcal {H}}^{*}\mathcal {H} \succeq 0, \end{aligned}$$

where $L:= \text {min}\{j,k\}$. As shown in Sect. 2.1, $\sqrt{L}$ is the upper bound of the spectral norm of the Hankel adjoint map $\mathcal {H}^{*}$.

Thus, given the variables $\mathbb {V}[i]$, the Lagrange multipliers $\mathbb {Y}[i]$ and the parameter $\mu [i]$ at iteration *i*, the updates of the proposed solver, summarized in Algorithm 1, are as follows.

*Update the Primal Variables*

15 $$\begin{aligned}&\mathbf {E}[i+1] = \mathop {{{\mathrm{arg\,min}}}}\limits _{ \mathbf {E} }{{\mathcal {L}}( \mathbf {E} ,\mathbb {Y}{[i]},\mu [i])} \nonumber \\&\quad = \mathop {{{\mathrm{arg\,min}}}}\limits _{ \mathbf {E} } \lambda {\mu [i]}^{-1} \left||{\mathbf {W}} \circ {\mathbf {E}} \right||^{q}_{q} \nonumber \\&\qquad + \frac{1}{2}\left||\mathbf {E} \!-\! \left( \mathbf {M} \!-\! \mathbf {L} \!+\! {\mu [i]}^{-1}{\varvec{\Lambda }_1}[i] \right) \right||^2_F \end{aligned}$$

16 $$\begin{aligned}&\mathbf {N}[i+1] \!=\! \mathop {{{\mathrm{arg\,min}}}}\limits _{ \mathbf {N} }{{\mathcal {L}}( \mathbf {N} ,\mathbb {Y}{[i]},\mu [i])} \nonumber \\&\quad = \mathop {{{\mathrm{arg\,min}}}}\limits _{ \mathbf {N} } {\mu [i]}^{-1}\left||{\mathbf {N}} \right||^{p}_{S_p} \nonumber \\&\qquad +\, \frac{1}{2}\left||\mathbf {N} - \left( \mathcal {H}(\mathbf {L}) - {\mu [i]}^{-1}{\varvec{\Lambda }_2}[i] \right) \right||^2_F \end{aligned}$$

17 $$\begin{aligned}&{\mathbf {L}}[i+1] \!=\! \mathop {{{\mathrm{arg\,min}}}}\limits _{{\mathbf {L}}}{\mathcal {L}}({\mathbf {L}},\mathbb {Y}{[i]},\mu [i])\!+\!\frac{\mu [i]}{2} \left||\mathbf {L}-\mathbf {L}[i] \right||^{2}_{\mathcal {Q}_0}\nonumber \\ \end{aligned}$$

*Update the Lagrange Multipliers*

18 $$\begin{aligned} {\varvec{\Lambda }_1}[i+1]&= {\varvec{\Lambda }_1}[i] + \mu [i] \left( \mathbf {M} - \mathbf {L} - \mathbf {E} \right) \end{aligned}$$

19 $$\begin{aligned} {\varvec{\Lambda }_2}[i+1]&= {\varvec{\Lambda }_2}[i] + \mu [i] \left( \mathbf {N} - \mathcal {H}(\mathbf {L}) \right) \end{aligned}$$

Equation (15), which offers the update for $\mathbf {E}$, is solved based on the *generalized soft thresholding operator* proposed in Nie et al. (2013) and briefly described next. Consider the following problem.

20 $$\begin{aligned} \mathop {{{\mathrm{arg\,min}}}}\limits _{ \mathbf {B} }{ \alpha \left||{\mathbf {B}} \right||^{q}_{q} + \frac{1}{2}\left||\mathbf {B} - \mathbf {Z} \right||^2_F}, \end{aligned}$$

with $\mathbf {B} \in \mathbb {R}^{m \times n}$ and $\alpha$ a positive parameter. Problem (20) is separable with respect to the elements of ${\mathbf {B}}$ and is thereby decomposed into $m \times n$ sub-problems of the form

21 $$\begin{aligned} \min _{ b_{ij} }{ \alpha \vert b_{ij} \vert ^q + \frac{1}{2}(b_{ij} - z_{ij} )^2}. \end{aligned}$$

Let us now define $h(b_{ij})=\alpha \vert b_{ij} \vert ^q + \frac{1}{2}(b_{ij} - z_{ij} )^2$, $c_1 = \left( \alpha q (1-q) \right) ^{\frac{1}{2-q}}$ and $c_2 = c_1 + \alpha q \vert c_1 \vert ^{q-1}$. Equation (21) admits an analytical solution for $q \in (0,1]$ given by

22 $$\begin{aligned} b^{*}_{ij} = {\left\{ \begin{array}{ll} 0 &{} \quad \text {if } \vert b_{ij} \vert \le c_2 \\ {{\mathrm{arg\,min}}}_{b_{ij} \in \{0, \rho _1 \}}{h(b_{ij})} &{} \quad \text {if } b_{ij} > c_2 \\ {{\mathrm{arg\,min}}}_{b_{ij} \in \{0, \rho _2 \}}{h(b_{ij})} &{} \quad \text {if } b_{ij} < -c_2, \end{array}\right. } \end{aligned}$$

where $\rho _1$ and $\rho _2$ are the roots of $h'(b_{ij})=\alpha q \vert b_{ij} \vert ^{q-1}\text {sgn}(b_{ij}) + b_{ij} - z_{ij} = 0$ in $[c_1, z_{ij}]$ and $[z_{ij}, -c_1]$, respectively. The roots can easily be found by applying the iterative Newton–Raphson root-finding method initialized at $z_{ij}$. Similarly to Papamakarios et al. (2014), we henceforth call the element-wise solver (22) *generalized q-shrinkage operator* and denote it by $\mathcal {S}^{q}_{\alpha }\{ \cdot \}$. Note that when $q=1$ the aforementioned operator reduces to the element-wise application of the well-known *shrinkage operator* (Candès et al. 2011), defined by

23 $$\begin{aligned} \mathcal {S}_{\alpha }\{ x \} := \text {sgn}(x)\text {max}\{\vert x \vert - \alpha ,0\}. \end{aligned}$$

We shall denote by $\mathcal {S}^{q}_{(\alpha ,{{\mathbf {W}}})}\{ \cdot \}$ the operator for which ${\bar{\alpha }}=\alpha w_{ij}$, with $\mathbf {W} \in \mathbb {R}^{m \times n}$ known, is used instead of $\alpha$ for the solution of each respective $b_{ij}$ in (22).

The solution of (16), that is, the minimization of (13) with respect to $\mathbf {N}$, is based on the following Lemma.

#### Lemma 1

(Nie et al. 2013) The solution of the optimization problem

24 $$\begin{aligned} \mathop {\mathop {{{\mathrm{arg\,min}}}}\limits }\limits _{\mathbf {B}}{ a \left||{\mathbf {B}} \right||^{p}_{S_p} + \frac{1}{2}\left||\mathbf {B} - \mathbf {Z} \right||^2_F}, \end{aligned}$$

with $p \in (0,1]$, is given by ${\mathbf {B}} = \mathbf {U_S}\mathcal {S}^{p}_{\alpha }\{ \varvec{\Sigma } \}\mathbf {V_{S}}^T$, where $\mathbf {U_S}\varvec{\Sigma }\mathbf {V_{S}}^T=\mathbf {Z}$ is the SVD of $\mathbf {Z}$.

We shall denote by $\mathcal {D}^{p}_{\alpha }\{ \cdot \}$ the operator – henceforth called *generalized singular value p-shrinkage operator* – that solves (24).

Clearly, problem (17) admits a closed-form solution.

The proposed ADMM-based solver is summarized in Algorithm 1. The latter is terminated when the following conditions are met

25 $$\begin{aligned} \scriptstyle \begin{Bmatrix} \begin{aligned}&\max \bigg \lbrace \frac{\left||\mathbf {M} - \mathbf {L}[i+1] - \mathbf { E}[i+1] \right||_{F}}{\left||\mathbf {M} \right||_{F}}, \\&\frac{\left||\mathbf {N}[i+1] - \mathbf {{\mathcal {H}}}(\mathbf {L}[i+1]) \right||_{F}}{\left||\mathbf {M} \right||_{F}}\bigg \rbrace< \epsilon _1 , \\&\max \bigg \lbrace \frac{\left||\mathbf {N}[i+1] - \mathbf {N}[i] \right||_{F}}{\left||\mathbf {M} \right||_{F}}, \frac{\left||\mathbf {L}[i+1] - \mathbf {L}[i] \right||_{F}}{\left||\mathbf {M} \right||_{F}}, \\&\frac{\left||\mathbf {E}[i+1] - \mathbf {E}[i] \right||_{F}}{\left||\mathbf {M} \right||_{F}} \bigg \rbrace < \epsilon _2, \end{aligned} \end{Bmatrix} \end{aligned}$$

where $\epsilon _1$ and $\epsilon _2$ are small positive parameters, or a maximum of 1000 iterations are reached.
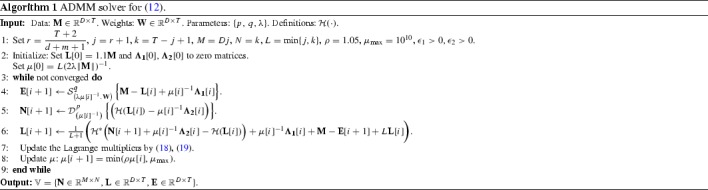


*Computational Complexity and Convergence* The cost of each iteration in Algorithm 1 is dominated by the calculation of the *generalized singular value p-shrinkage operator* in Step 5, which involves a complexity equal to that of SVD, i.e., ${\mathcal {O}}\left( \max \{M^2N, MN^2\}\right)$. The *generalized q-shrinkage operator*, utilized in Step 4, entails linear complexity ${\mathcal {O}}(DT)$.

Regarding the convergence of Algorithm 1, there is no established convergence proof of the ADMM for problems in the form of (12). Indeed, the ADMM is only known to converge for convex separable problems with up to two blocks of variables (e.g., Bertsekas 2014; Candès et al. 2011). However, this is not the case even in the convex instance of (12) (i.e., when $p=q=1$), since the optimization problem involves more than two blocks of variables. For the multi-block separable convex problems, with three or more blocks of variables, it is known that the original ADMM is not necessarily convergent (Chen et al. 2016). On the other hand, theoretical convergence analysis of the ADMM for non-convex problems is rather limited, making either assumptions on the iterates of the algorithm (Xu et al. 2012; Magnusson et al. 2016) or dealing with special non-convex models (Li and Pong 2015; Wang et al. 2014a, 2015), none of which is applicable for the proposed optimization problem (12). However, it is worth noting that the ADMM exhibits good numerical performance in non-convex problems such as non-negative matrix factorization (Sun and Févotte 2014), tensor decomposition (Liavas and Sidiropoulos 2015), matrix separation (Shen et al. 2014; Papamakarios et al. 2014), matrix completion (Xu et al. 2012), motion segmentation (Li et al. 2014), to mention but a few.

To the best of our knowledge, the only work which focuses on the convergence analysis of the ADMM when applied for the optimization of piecewise linear functions such as the Schatten *p*-norm and the $\ell _q$-norm (when $0 < p,\, q \le 1$) is the recent preprint of Wang et al. (2016). However, since a systematic convergence analysis is out of the scope of this paper, we plan to adapt the analysis in Wang et al. (2016) in order to analyze the convergence of the proposed algorithm in the future.

**Fig. 2:**
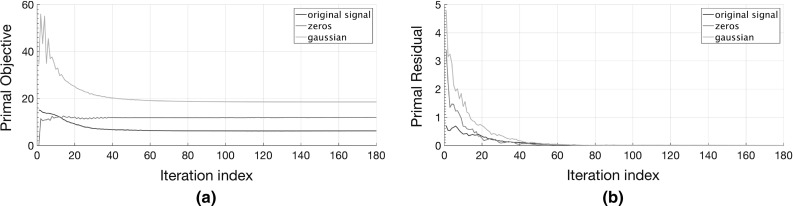
(Better viewed in *color*). Empirical convergence analysis results for three different initializations of the proposed solver [Algorithm 1 with $(p,\,q) = (0.5,0.5)$] illustrated for the reconstruction of synthetic data corresponding to system order $n=6$. The graphs illustrated are plots of the value of **a** the *Primal Objective* $\left||\mathbf {N} \right||^{p}_{S_p} + \lambda \left||\mathbf {E} \right||^{q}_{q}$, and **b** the *Primal Residual* $\Vert \mathbf {M} - \mathbf {L}-\mathbf {E} \Vert _F$ of the proposed method (12), with the iteration index. Note that $\mathbf {M}={\tilde{\mathbf {y}}}$ denotes the given noisy data and $\mathbf {L}={\hat{\mathbf {y}}}$ the reconstruction in this experiment. The different initializations of the matrix $\mathbf {L}$ in Algorithm 1 correspond to the following scenarios: {‘multiple’: $\mathbf {L}[\mathbf {0}] = 1.1{\tilde{\mathbf {y}}}$, ‘zeros’: $\mathbf {L}[\mathbf {0}] = \mathbf {0}$, ‘gaussian’: $\mathbf {L}[\mathbf {0}][t] \sim {\mathcal {N}}(0,1), \; t=1,2,\ldots ,T$ (mean value over 10 repetitions)}, where *T* denotes the number of observations (Color figure online)

Even though we cannot theoretically guarantee the convergence of the proposed solver, the experimental results on synthetic data in Sect. 6.1 show that its numerical performance is good in practice. Specifically, the empirical convergence of the proposed solver is evidenced, where both the primal residual and the primal objective are non-increasing after the very few iterations (see Fig. 2). Similar convergence behavior characterizes also the experiments on real-world data presented in Sect. 6, where we have observed that even the non-convex variant with $p=q=0.1$ of the proposed method (12) needs no more than 180 iterations to converge in most cases.

### Scalable Version of the Algorithm

To improve the scalability and reduce the computational complexity of the ADMM-based Algorithm 1, we develop here a scalable version. Depending on the application, and more specifically, the number of inputs and/or outputs employed and the number of observations, the dimension of the Hankel matrix $\mathcal {H}(\mathbf {L}) \in \mathbb {R}^{M \times N}$ can rise largely, which makes the calculation of SVD prohibitive. To alleviate the aforementioned computational complexity issue, we further impose that $\mathcal {H}(\mathbf {L}) \in \mathbb {R}^{M \times N}$ is factorized into an orthonormal matrix and a low-rank matrix as $\mathcal {H}(\mathbf {L})=\mathbf {QR}$, with $\mathbf {Q} \in \mathbb {R}^{M \times K}$, $\mathbf {R} \in \mathbb {R}^{K \times N}$ and $K \ll M, N$. In this factorization, $\mathbf {Q} \in \mathbb {R}^{M \times K}$ is a column-orthogonal matrix satisfying $\mathbf {Q}^T\mathbf {Q} = \mathbf {I}$ and $\mathbf {R} \in \mathbb {R}^{K \times N}$ is a low-rank matrix representing the embedding of $\mathcal {H}(\mathbf {L})$ onto the *K*-dimensional subspace spanned by the columns of $\mathbf {Q}$.

Due to the unitary invariance of the Schatten *p*-norm, the following equality holds $\left||\mathbf {QR} \right||_{S_p} = \left||{\mathbf {R}} \right||_{S_p}$. Thus, by incorporating the factorization $\mathcal {H}(\mathbf {L})=\mathbf {QR}$ and adding the orthonormality constraint for $\mathbf {Q}$, (12) is written as

26 $$\begin{aligned} \begin{aligned}&\min _{{\mathbf {R}},{\mathbf {L}},{\mathbf {E}},{\mathbf {Q}}} \; {\left||{\mathbf {R}} \right||^{p}_{S_p} + \lambda \left||{\mathbf {W}} \circ {\mathbf {E}} \right||^{q}_{q}} \\&\text {s.t.} \quad \begin{Bmatrix} \begin{aligned}&\mathbf {M} = \mathbf {L}+\mathbf {E}, \\&\mathbf {QR} = \mathcal {H}(\mathbf {L}), \\&{\mathbf {Q}}^\top {\mathbf {Q}} = {\mathbf {I}}. \end{aligned} \end{Bmatrix} \end{aligned} \end{aligned}$$

Since $M K + K N \ll M N$, the number of variables has been significantly reduced. Clearly, this modification reduces the overall complexity of the method, since the SVD is now applied on $M \times K$ and $K \times N$ matrices as opposed to a $M \times N$ matrix.

The ADMM is employed to solve (26). With $\mathbb {V}:=\{{\mathbf {R}} \in \mathbb {R}^{K \times N},{\mathbf {L}} \in \mathbb {R}^{D \times T},{\mathbf {E}} \in \mathbb {R}^{D \times T}, {\mathbf {Q}} \in \mathbb {R}^{M \times K}\}$ and $\mathbb {Y}:=\{{\varvec{\Lambda }_1} \in \mathbb {R}^{D \times T},{\varvec{\Lambda }_2} \in \mathbb {R}^{M \times N}\}$ defined as the sets containing all the unknown variables and the Lagrange multipliers for the first two equality constraints in (26), respectively, the (partial) augmented Lagrangian function is defined as

27 $$\begin{aligned} \begin{aligned} \displaystyle&\mathcal {L^{\text {sc}}}({\mathbb {V},\mathbb {Y},\mu }) \, = \left||{\mathbf {R}} \right||^{p}_{S_p}+\lambda \left||{\mathbf {W}} \circ {\mathbf {E}} \right||^{q}_{q} \\&\quad +\left\langle {\mathbf {M} - \mathbf {L}-\mathbf {E},{\varvec{\Lambda }_1}}\right\rangle +\left\langle {\mathbf {QR} - \mathcal {H}(\mathbf {L}),{\varvec{\Lambda }_2}}\right\rangle \\&\quad + \frac{\mu }{2}\Big ( \left|| \mathbf {M} - \mathbf {L}-\mathbf {E} \right||^2_F +\left|| \mathbf {QR} - \mathcal {H}(\mathbf {L}) \right||^2_F \Big ), \end{aligned} \end{aligned}$$

where $\mu$ is a positive parameter. Therefore, at each iteration of the ADMM-based solver for (26), we solve

28 $$\begin{aligned} \min _{\mathbb {V}}{\mathcal {L^{\text {sc}}}({\mathbb {V},\mathbb {Y},\mu })} \quad \text {s.t.} \quad {\mathbf {Q}}^\top {\mathbf {Q}} = {\mathbf {I}}, \end{aligned}$$

with respect to each variable in $\mathbb {V}$ in an alternating fashion and, subsequently, the Lagrange multipliers in $\mathbb {Y}$ and the parameter $\mu$ are updated.

The proposed solver for (26) is summarized in Algorithm 2. The updates for $\mathbf {R},\mathbf {L},\mathbf {E}$ are similar to those employed to solve (12). The solution of (28) with respect to $\mathbf {Q}$ is based on the *Procrustes operator*, which is defined as ${\mathcal {P}}[{\mathbf {L}}] = {\mathbf {A}}{\mathbf {B}}^{T}$ for a matrix $\mathbf {L}$ with SVD ${\mathbf {L}} = {\mathbf {A}}\varvec{\Sigma }{\mathbf {B}}^{T}$ and solves the problem in the following Lemma.

#### Lemma 2

(Zou et al. 2006) The constrained minimization problem:

29 $$\begin{aligned} \mathop {{{\mathrm{arg\,min}}}}\limits _{{\mathbf {B}}}{\Vert {\mathbf {A}} - {\mathbf {B}}\Vert ^{2}_{F} \quad \text {s.t.} \quad {\mathbf {B}}^{T}{\mathbf {B}} ={\mathbf {I}}} \end{aligned}$$

has a closed-form solution given by ${\mathbf {P}} = {\mathcal {P}}[{\mathbf {A}}]$.


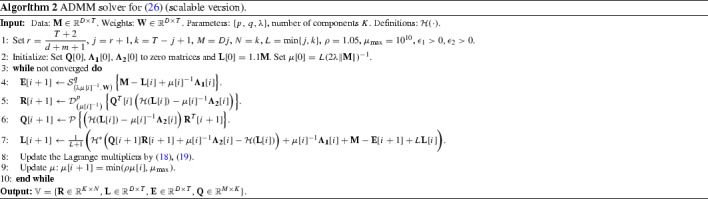


*Computational Complexity and Convergence* The cost of each iteration in Algorithm 2 is dominated by the calculation of the *generalized singular value p-shrinkage operator* and the *Procrustes operator* in Step 5 and 6, respectively, which both rely on SVD, thus involving respective complexities of ${\mathcal {O}}\left( \max \{K^2N, KN^2\}\right)$ and ${\mathcal {O}}\left( \max \{M^2K, MK^2\}\right)$. It is worth stressing again that choosing $K \ll M, N$, which implies $M K + K N \ll M N$, leads to a significantly reduced overall complexity for Algorithm 2 compared to that of Algorithm 1, which is instead dominated by a SVD on a $M \times N$ matrix, hence ${\mathcal {O}}\left( \max \{M^2N, MN^2\}\right)$. Again, the * generalized q-shrinkage operator*, utilized in Step 4, entails linear complexity ${\mathcal {O}}(DT)$.

Regarding the convergence of Algorithm 2 which solves the scalable version of the proposed model (26), there is no yet established convergence proof of the ADMM for problems in the form of (26). The discussion provided above on the convergence of Algorithm 1 applies to a large extent for Algorithm 2 as well. As a matter of fact, theoretical analysis for the convergence of Algorithm 2 becomes more challenging, compared to the case of Algorithm 1, considering that the factorization $\mathbf {QR} = \mathcal {H}(\mathbf {L})$ and the non-linear orthonormality constraint ${\mathbf {Q}}^\top {\mathbf {Q}} = {\mathbf {I}}$ are introduced in the scalable version of the proposed model (26). It is also worth noting that problem (26) is always non-convex due to these two equality constraints, and thus the solutions yielded by the optimization problems (12) and (26) cannot be related. However, it has been shown in Liu and Yan (2012) that the ADMM converges to a local minimum for a problem similar to problem (26) with convex objective function, i.e., $p,\,q \ge 1$. To the best of our knowledge, for the case $0< p,\,q < 1$, i.e., when the Schatten *p*-norm and the $\ell _q$-norm act as non-convex approximations of the rank function and the $\ell _0$-(quasi) norm, respectively, there has been no theoretical evidence for the convergence of the ADMM for the problem (26) and further investigation is needed.

Nevertheless, the ADMM has been shown to achieve good numerical performance in non-convex subspace learning problems employing a similar matrix factorization approach with one of the factors being orthonormal (Sagonas et al. 2014; Papamakarios et al. 2014). Also, experimental results on synthetic data evidence the empirical convergence of Algorithm 2, which has been found to be similar to that shown for Algorithm 1 ($p=q=0.5$) in Fig. 2. Good numerical performance is also achieved by the scalable solver in the experiments presented in Sect. 6.

## Dynamic Behavior Analysis Frameworks based on Hankel Structured Rank Minimization

In this section, we develop two frameworks for dynamic behavior analysis.

### Dynamic Behavior Prediction

Consider the case where continuous-time, real-valued annotations characterizing dynamic behavior or affect (e.g., conflict, valence, arousal), manifested in a video sequence of *T* frames, are available for a number of consecutive frames $t=0,1,\ldots ,T_{train}-1$ (training set). The goal herein is to first learn a low-order LTI system that generates the annotations as outputs $\mathbf {Y} = [\mathbf {y_0}, \mathbf {y_1}, \ldots , \mathbf {y_{T_{train}-1}}] \in \mathbb {R}^{m \times T_{train}}$ when visual features act as inputs $\mathbf {U} = [\mathbf {u_0}, \mathbf {u_1}, \ldots , \mathbf {u_{T_{train}-1}}] \in \mathbb {R}^{d \times T_{train}}$, and next use it to predict behavior measurements $\hat{\mathbf {y}_t}$ for the remaining frames of the sequence $t = T_{train},\ldots ,T-1$ (test set), based on the respective features $\mathbf {u_t}$. To this end, the following framework is proposed.

First, the proposed structured minimization problem (10) is solved, with $\mathbf {M}=\mathbf {Y}$ and the Hankel map ${\mathcal {H}}(\cdot )$ defined as in Sect. 2.2, to estimate the system order. Second, the low-rank solution $\mathcal {H}(\mathbf {L})$ is used to estimate the system matrices ${\hat{\mathbf {A}},\hat{\mathbf {B}},\hat{\mathbf {C}},\hat{\mathbf {D}}}$ and the initial state vector ${\hat{\mathbf {x}}_0}$ by solving a system of linear equations, following, for example, Van Overschee and De Moor (2012). Finally, test set predictions ${\hat{\mathbf {y}}}$ ($t = T_{train},\ldots ,T-1$) for dynamic behavior are obtained by applying the equations of the learned state-space model (3) for $t=0,1,\ldots ,T_{train}-1$, with the visual features used as inputs $\mathbf {u_t}$.

*Applications* The aforementioned framework can be used for continuous prediction of any number or type of real-valued behavioral attributes manifested in a video sequence, by employing a portion of consecutive frames (even a few seconds) to learn a LTI system as described above (see Sect. 6).

### Dynamic Behavior Prediction with Partially Missing Outputs

Consider now the scenario in which the goal is to predict *missing* (or unreliable) and not necessarily consecutive real-valued measurements of dynamic behavior or affect, viewed as missing outputs ${\bar{\mathbf {y}}_t}$ of a low-order LTI system, directly by employing the observed visual features as inputs $\mathbf {u_t}$ and the available annotations as outputs $\mathbf {y_t}$, without explicitly learning the system. Herein, we approach this task as a (Hankel) structured low-rank *matrix completion* problem and address it by means of the following predictive framework that is based on the proposed model (12).

Let $\mathbf {Y} = [\mathbf {y_0}, \mathbf {y_1}, \ldots , \mathbf {y_{T-1}}] \in \mathbb {R}^{m \times T}$ and $\mathbf {U} = [\mathbf {u_0}, \mathbf {u_1}, \ldots , \mathbf {y_{T-1}}] \in \mathbb {R}^{m \times T}$ be the matrices containing all *T* observations (available and missing) of inputs and outputs, respectively, and let $$ \mathbf {M} = \begin{bmatrix} \mathbf {Y} \\ \mathbf {U} \end{bmatrix} \in \mathbb {R}^{D \times T} $$ and $$ \mathcal {H}(\mathbf {\mathbf {M}}) = H_{D,1,r+1,T-r}\left( \begin{bmatrix} \mathbf {Y} \\ \mathbf {U} \end{bmatrix} \right) $$, with $D=m+d$. Let also $\Omega \subset [1,D] \times [1,T]$ be the set containing the indices of the observed (available) entries in $\mathbf {M}$. When outputs are noisy, the following property holds only approximately (Van Overschee and De Moor 2012), under the assumption of persistently exciting inputs.

30 $$\begin{aligned} rank \left( \mathcal {H}(\mathbf {\mathbf {M}}) \right) = n + rank \left( H(\mathbf {U}) \right) . \end{aligned}$$

Thus, a low-rank approximation of $\mathcal {H}(\mathbf {M})$ should be obtained to estimate the true order of the system *n*.

To this end, the proposed model (12) is solved, with $\mathbf {M}$ defined as above and $\mathbf {W}$ computed according to (11). Note that this process simultaneously ‘completes’ the missing observations of $\mathbf {M}$, by forcing the approximation of $\mathcal {H}(\mathbf {M})$ to be low-rank, or in other words, the ‘completed’ trajectory $\mathbf {L}$ to follow the same linear dynamics underlying the observed trajectory $\mathbf {M}$. Finally, the missing outputs are recovered from the respective entries of the low-rank approximation $\mathcal {H}(\mathbf {L})$. Notably, this framework has the advantage that the missing observations are obtained directly by solving  (12), thus avoiding the computational load associated with learning a minimum order realization of the system.

*Applications* The aforementioned framework can achieve prediction of missing (past or future) observations pertaining to dynamic human behavior or affect, with the latter used as outputs of a low-order LTI system. For instance, a computer vision problem that can be addressed by means of the proposed framework is the problem of *tracklet matching* (Ding et al. 2007a, 2008; Dicle et al. 2013), which consists of stitching trajectories of detections belonging to the same target. For this task, one needs to assess whether the joint trajectory of detections $$ \mathbf {M} = \begin{bmatrix} \mathbf {Y_{start}} \bar{\mathbf {Y}}_{\mathbf {{inter}}} \mathbf {Y}_{\mathbf {end}}\end{bmatrix} $$, where $\mathbf {Y_{start}}$ and $\mathbf {Y_{end}}$ are the observed trajectories and $\bar{\mathbf {Y}}_{\mathbf {{inter}}}$ is a zero-valued matrix corresponding to the ‘missing’ intermediate trajectory, is the output of the same autonomous (output-only) LTI system that generated $\mathbf {Y_{start}}$ and $\mathbf {Y_{end}}$. This is achieved by solving (12) for $\bar{\mathbf {Y}}_{\mathbf {{inter}}}$, with $\mathbf {M}$ defined as above, and subsequently comparing $\text {rank}(\mathcal {H}(\mathbf {L}))$ with $\text {rank}(\mathcal {H}(\mathbf {\mathbf {Y_{start}}}))$ and $\text {rank}(\mathcal {H}(\mathbf {\mathbf {Y_{end}}}))$ (see Sect. 6.4).

## Experiments

The efficiency of the proposed structured rank minimization methods is evaluated on synthetic data corrupted by sparse, non-Gaussian noise (Sect. 6.1), as well as on real-world data with applications to: (i) *conflict intensity prediction* (Sect. 6.2), (ii) *valence–arousal prediction* (Sect. 6.3), and (iii) *tracklet matching* (Sect. 6.4). For the case of dynamic behavior analysis experiments on real-world data, for the first two tasks, the framework described in Sect. 5.1 is employed, while for the last we utilize the framework described in Sect. 5.2.

Aside from the proposed methods, five structured minimization methods are also examined, namely HRM^2^ (Fazel et al. 2013), SVD-free (Signoretto et al. 2013), SRPCA (Ayazoglu et al. 2012), IHTLS (Dicle et al. 2013), and SLRA (Markovsky 2014) (see further details on these methods in Table 1). For all experiments presented in our paper, a grid search is employed to tune the parameter $\lambda$ of the proposed methods or any other parameters of the compared methods that need tuning. Tuning is performed by following an *out-of-sample evaluation*, that is, the last portion of the training frames is withheld for validation and the best-performing model is used for testing. Specifically, the last 2*r* training observations, with *r* defined in Sect. 3, are kept out for validation in all our experiments.

### Experiment on Synthetic Data

In the experiments presented in this section, the efficiency of the proposed method (12) is evaluated on synthetic data corrupted with sparse, non-Gaussian noise. In order to generate Hankel matrices of given rank *n*, we follow the methodology proposed in Park et al. (1999), that is, *T* outputs *y*(*t*) of an autonomous stable LTI system of order *n* are generated by applying the following formula

31 $$\begin{aligned} y(t) = \sum _{k=1}^{n}{z_k^{t}}, \quad t=1,2,\ldots ,T, \end{aligned}$$

where $$z_k $$ appear in pairs of conjugate numbers so that the observations *y*(*t*) are real numbers. It follows naturally that a $$M \times N$$ Hankel matrix $$\mathbf {Y} = \mathcal {H}(\mathbf {y}) = H_{1,1,M,N}(\mathbf {y})$$ with $$\mathbf {y}$$ derived according to (31) has rank equal to *n* (Park et al. 1999). Subsequently, sparse, non-Gaussian noise $$\varvec{\eta } \in \mathbb {R}^{1 \times T}$$ is added to the original signal $$\mathbf {y}$$, with the non-zero entries following the Bernoulli model with probability $$\rho =0.2$$, as in Candès et al. (2011). The final corrupted signal is formed as $$\tilde{\mathbf {y}} = \mathbf {y} + \varvec{\eta }$$, with the corresponding noisy Hankel matrix $$\tilde{\mathbf {Y}} = \mathcal {H}({\tilde{{\mathbf {y}}}})$$ being full-rank.

In what follows, the efficiency of various structured rank minimization methods in reconstructing the noiseless system outputs *y*(*t*), $t =1,2,\ldots ,T$, by finding a low-rank approximation ${\hat{\mathbf {Y}}}=\mathcal {H}({\hat{\mathbf{y}}})$ given the noisy Hankel matrix ${\tilde{\mathbf {Y}}}$, is experimentally assessed in various scenarios.

The reconstruction error, for both the noiseless observations $\mathbf {y}$ and the noise $\varvec{\eta }$, is measured in terms of relative reconstruction error as follows.

32 $$\begin{aligned} \text {err}(\mathbf {s},{\hat{\mathbf {s}}}) = \frac{\Vert \mathbf {s} - {\hat{\mathbf {s}}} \Vert }{\Vert \mathbf {s} \Vert }, \end{aligned}$$

with $\mathbf {s}$ denoting the original signal and ${\hat{\mathbf {s}}}$ denoting the estimated signal by the algorithm.

*Experiment with Varying System Orders* Herein, experiments are conducted for various orders of the LTI system generating the ‘clean’ data, as described above. Specifically, the system order *n* is varied in $\{6,12,18 \}$. For each value of *n* the experiment is repeated 10 times, that is, for 10 different output trajectories $\mathbf {y} \in \mathbb {R}^{1 \times T}$ computed by randomly selecting the complex coefficients in (31). For the proposed model, Algorithm 1 is used and the following combinations are examined for the *p* and *q* values corresponding to the Schatten *p*- and $\ell _q$-norm, respectively: $(p,\,q) \in \{(1, 1), (0.9, 0.9), (0.5, 0.5), (0.1, 0.1)\}$. The methods HRM, SVD-free, SRPCA, IHTLS, and SLRA (listed in Table 1) are also evaluated for comparison. For each method, results are reported in terms of minimum reconstruction error $\text {err}(\mathbf {y},{\hat{\mathbf {y}}})$ computed according to (32). Performance is also evaluated in terms of reconstruction error for the noise signal $\text {err}(\varvec{\eta },\hat{\varvec{\eta }})$ and the Pearson Correlation Coefficient (COR) measured between the noiseless observations $\mathbf {y}$ and the reconstructed outputs ${\hat{\mathbf {y}}}$.

**Table 2:** Recovery results obtained by the proposed method and the compared methods corresponding to system order (a) $n=6$, (b) $n=12$ and (c) $n=18$

Method	$\text {err}\,(\mathbf {y},{\hat{\mathbf {y}}})$	$\text {err}\,(\varvec{\eta },\hat{\varvec{\eta }})$	**COR**	Order	Iter	Time
(a) System order $n=6$						
HRM	0.630 (0.161)	0.259 (0.119)	0.773 (0.145)	8 (2.3)	49 ( 32)	0.008 ( 0.005)
SVD-free	0.894 (0.181)	0.365 (0.167)	0.809 (0.169)	1 (0.4)	905 (301)	0.448 (0.162)
SRPCA	0.922 (0.142)	0.372 (0.137 )	0.677 (0.492)	7 (2.1)	101 (16)	0.030 (0.004)
IHTLS	0.629 (0.301)	0.267 (0.177)	0.810 (0.203)	2 (0.5)	41 (42)	0.011 (0.011)
SLRA	0.612 (0.292)	1.094 (0.085)	0.816 (0.190)	1 (0.5)	33 (23)	0.002 (0.002)
ours ($p=1$, $q=1$)	0.395 (0.218 )	0.173 (0.137)	0.900 (0.093)	6 (2.2)	90 (10)	0.016 (0.002)
ours ($p=0.9$, $q=0.9$)	0.313 (0.232)	0.141 (0.136)	0.926 (0.079)	5 (3.2)	130 (17)	0.026 (0.003)
ours ($p=0.5$, $q=0.5$)	0.299 (0.220)	0.129 (0.141)	0.933 (0.066)	6 (2.7)	215 (90)	0.047 (0.014)
ours ($p=0.1$, $q=0.1$)	**0.233 (0.218)**	**0.107 (0.132)**	**0.952 (0.061)**	5 (1.8)	217 (19)	0.043 (0.004)
(b) System order $n=12$						
HRM	0.692 (0.234 )	0.205 (0.097)	0.637 (0.352)	10 (7.5)	57 (31)	0.022 (0.012)
SVD-free	0.942 (0.104)	0.273 (0.077)	0.634 (0.343)	2 (0.7)	703 (478 )	0.544 (0.378)
SRPCA	0.655 (0.211)	0.181 (0.051)	0.848 (0.167)	6 (2.6)	102 (7)	0.064 (0.004)
IHTLS	0.719 (0.299)	0.217 (0.120)	0.616 (0.35)9	1 (0.5)	50 (43)	0.042 (0.030)
SLRA	0.832 (0.355)	1.071 (0.060)	0.416 (0.500)	1 (0.4)	58 (40)	0.006 (0.005)
ours ($p=1$, $q=1$)	0.414 (0.333)	0.120 (0.096)	0.813 (0.278)	6 (3.1)	107 (4)	0.042 (0.002)
ours ($p=0.9$, $q=0.9$)	0.365 (0.338)	0.103 (0.097)	0.856 (0.213)	6 (1.8)	148 (8)	0.063 (0.004)
ours ($p=0.5$, $q=0.5$)	**0.333 (0.363)**	**0.094 (0.105)**	**0.863 (0.199)**	5 (2.2)	210 (24)	0.089 (0.011)
ours ($p=0.1$, $q=0.1$)	0.341 (0.298)	0.111 (0.094)	0.859 (0.250)	13 (3.0)	181 (91)	0.088 (0.047)
(c) System order $n=18$						
HRM	0.780 (0.238)	0.216 (0.108)	0.483 (0.364)	8 (8.9)	87 (39)	0.063 (0.031)
SVD-free	0.889 (0.203)	0.242 (0.107)	0.567 (0.301)	1 (0.5)	619 (493)	0.789 (0.648)
SRPCA	0.626 (0.238)	0.160 (0.065)	0.752 (0.247)	8 (3.7)	107 (10)	0.127 (0.023)
IHTLS	0.945 (0.309)	0.247 (0.093)	0.479 (0.390)	2 (1.6)	41 ( 36)	0.082 (0.056 )
SLRA	0.958 (0.263)	1.082 (0.057)	0.471 (0.354)	2 (3.1)	65 (39)	0.012 (0.009)
ours ($p=1$, $q=1$)	0.572 (0.312)	0.151 (0.088)	0.723 (0.269)	6 (4.7)	108 (10)	0.076 (0.009)
ours ($p=0.9$, $q=0.9$)	0.552 (0.322)	0.144 (0.087)	0.736 (0.273)	6 (3.0)	154 (8)	0.133 (0.028)
ours ($p=0.5$, $q=0.5$)	0.534 (0.327)	0.141 (0.088)	0.739 (0.239)	6 (3.0)	154 (8)	0.133 (0.028)
ours ($p=0.1$, $q=0.1$)	**0.524 (0.346)**	**0.135 (0.091)**	**0.744 (0.241)**	6 (4.1)	223 (9)	0.171 (0.021)

The bold values indicate the best performances in terms of each evaluation metric

Results are reported in terms of mean values over 10 repetitions of the experiment, while standard deviation values are reported inside parentheses

Table 2a–c contain the results obtained by the various methods for system order $n=6$, $n=12$ and $n=18$, respectively. Specifically, mean and standard deviation values of the reconstruction errors $\text {err}(\mathbf {y},{\hat{\mathbf {y}}})$ and $\text {err}(\varvec{\eta },\hat{\varvec{\eta }})$ and the COR values computed over the 10 trials of each experiment are reported. The mean values of the estimated system order (rank of ${\hat{\mathbf {Y}}} = \mathcal {H}({\hat{\mathbf{y}}}$)), number of iterations and execution time are also reported.

Firstly, we observe that the non-convex instances of the proposed method, i.e., when $p,\,q < 1$, consistently account for the most accurate reconstruction of both the clean signal, in terms of both reconstruction error and correlation, as well as the recovery of the sparse noise. In most cases, the performance is improved when smaller values for *p* and *q* are chosen for the proposed model. Secondly, all the compared methods (HRM, SVD-free, SRPCA, IHTLS and SLRA) achieve much lower performance in terms of all the three metrics employed. Furthermore, it is worth noting that, in the scenarios corresponding to orders $n=12$ and $n=18$, SRPCA recovers the noise more accurately than the HRM, SVD-free, IHTLS and SLRA. This is expected since the former is the only method amongst the compared ones that is robust to sparse, non-Gaussian noise. It is also worth mentioning that the system order pertaining to the recovered observations varies significantly amongst different methods. Amongst the different instances of the proposed method, this variation is much smaller, with the only exception being the result obtained by our method with $(p,\,q) = (0.1, 0.1)$ for the case $n=12$. Regarding the number of iterations, which varies largely across methods, we observe that the non-convex instances of the proposed method require a larger amount of iterations to converge, as compared to the convex instance ($p=q=1$). However, even in the scenario of order $n=18$, the best-performing instance of the proposed method ($p=q=0.1$) needs 223 iterations in average to converge. Finally, the execution times corresponding to the best-performing, non-convex instances of the proposed method in all three experiments are comparable to those accounted for by even convex compared methods, such as SRPCA.

*Empirical Convergence Analysis* In this experiment, the convergence of the proposed method is assessed by employing various types of initialization. To this end, we employ synthetic data corrupted with sparse, non-Gaussian noise, generated similarly to the previous experiment. We clarify here that the only variable that needs to be initialized in Algorithm 1, except for the Lagrange multipliers, is the matrix $\mathbf {L}$. All other variables are calculated in the 1st iteration of the ADMM loop according to the respective updates.

The proposed solver is executed using the following three types of initialization, namely, ‘original signal’: $\mathbf {L}[\mathbf {0}] = 1.1{\tilde{\mathbf {y}}}$, ‘zeros’: $\mathbf {L}[\mathbf {0}] = \mathbf {0}$, ‘gaussian’: $\mathbf {L}[\mathbf {0}][t] \sim {\mathcal {N}}(0,1), \; t=1,2,\ldots ,T$, where ${\tilde{\mathbf {y}}}$ denote the noisy system outputs constructed as in the previous experiments and ${\mathcal {N}}(0,1)$ denotes the normal distribution. For each type of initialization, the values of the *primal objective* ($\left||\mathbf {N} \right||^{p}_{S_p} + \lambda \left||\mathbf {E} \right||^{q}_{q}$) and the *primal residual* ($\Vert \mathbf {M} - \mathbf {L}-\mathbf {E} \Vert _F$ ) of the proposed model (12) are plotted as a function of the iteration index in Fig. 2. Here $\mathbf {M}={\tilde{\mathbf {y}}}$ denotes the given noisy data and $\mathbf {L}={\hat{\mathbf {y}}}$ the reconstruction. These plots enable us to demonstrate the convergence of the proposed solver. Note that for the last initialization scenario, the experiment is repeated 10 times. and the average convergence curve is plotted.

By inspecting both graphs, it is evident that all three initializations lead to similar convergence behavior in the sense that both the primal objective and the primal residual are non-increasing after the first few iterations. However, by initializing the algorithm using the scaled version of the original signal ($\mathbf {L}[\mathbf {0}] = 1.1{\tilde{\mathbf {y}}}$) the primal objective attains smaller values than the other two types of initialization. This justifies our choice of initialization as $\mathbf {L}[\mathbf {0}] = 1.1{\tilde{\mathbf {y}}}$ in the proposed algorithms.

### Conflict Intensity Prediction

In this section, we address the problem of continuous *conflict* intensity prediction based on the visual modality only. Conflict is usually defined as disagreement of high intensity or escalation, in which at least one of the involved interlocutors feels emotionally offended (Bousmalis et al. 2009). Hence, various challenges are posed to machine analysis of conflict in real-world competitive conversations, since simultaneous processing of the data streams from all interactants is required. Furthermore, when the visual modality is also considered, feature extraction has to cope with various types of visual noise, such as extreme head pose values and abrupt body movements, which renders computer vision pre-processing (e.g., tracking, alignment) rather difficult.

**Fig. 3:**
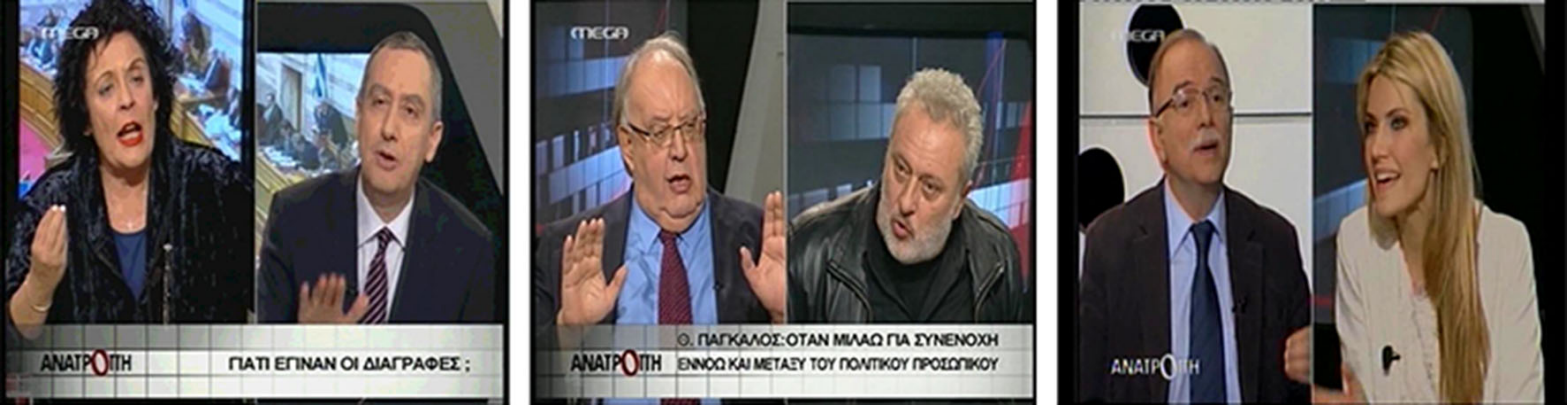
Three sample snapshots from the CONFER dataset, corresponding to dyadic conversations of two guests in conflict

Automated approaches to conflict analysis include just a few works, which are based on audio features only (Kim et al. 2012a, b). However, visual features can help discover facial behavioral cues that are intrinsically correlated with conflict, such as smiling, blinking, head nodding, flouncing and frowning. The only audio-visual approach to conflict detection that we are aware of is Panagakis et al. (2016), where robust, multi-modal fusion of audio-visual cues is utilized. However, all works mentioned above address conflict or conflict escalation detection within a classification framework predicting binary (conflict/non-conflict) or discretized conflict intensity labels.

To the best of our knowledge, the presented experiments constitute the first work that (i) addresses *continuous* conflict intensity prediction through a dynamic modeling framework (as opposed to frame-by-frame classification or regression), and (ii) uses *visual features only*.

*Data* In view of the absence of benchmark datasets for conflict detection, video excerpts from live political debates, aired on Greek television^3^ in between 2011 and 2012, are utilized. It is worth stressing that these debates, despite being moderated by the TV host, include unscripted dyadic interactions which are highly likely to lead to real conflict due to the participants acting under incompatible motives and interests. From the entire dataset, 160 audio-visual non-overlapping recordings with total duration amounting to 170 mins, have been manually extracted. These videos have been annotated by 10 experts, all of them being native Greek speakers, in terms of continuous conflict intensity. The temporal resolution of the video stream is 25 frames per second. Only the episodes involving exactly two interlocutors (97 out of 160 samples) are considered herein. For each sequence, the mean over the 10 available ratings, normalized to [0, 1], is used as ground truth for conflict intensity. Three sample snapshots from the dataset, henceforth called Conflict Escalation Resolution Database (CONFER), are depicted in Fig. 3.

*Features and Experimental Protocol* For visual feature extraction, we use the Gauss-Newton Deformable Part Model in Tzimiropoulos and Pantic (2013) for facial landmark detection, which when combined with a person-specific face detector produces very accurate results (Chrysos et al. 2015), to detect 49 fiducial facial points in each frame of an input video for each of the two interactants. The points are subsequently globally registered, using a 2-D non-reflective similarity transformation with respect to 4 reference points (centers of the eyes, center of the nose and top of the nose), to remove the effects of head translation, scale and in-plane rotation. This way, *yaw* and *pitch* pose angles, which are expected to be informative in terms of conflict, are retained in the shape configuration. Finally, Principal Component Analysis (PCA) is used at each frame to reduce dimensionality for the points of each speaker to 7, based on the components collectively accounting for 98% of the total variance.

The dynamic behavior prediction framework described in Sect. 5.1 is applied separately for each sequence used in the experiments of this section. During training, the stacked feature vectors corresponding to the two interlocutors are used as inputs $\mathbf {u_t}$ at each time frame *t* of the training set ($t \in [0,T_{train}-1]$), while the ground truth is used as output $\mathbf {y_t}$ of a LTI system. The goal is to predict the output ${\hat{\mathbf {y}}_t}$ (conflict intensity) for each frame of the sequence ($t \in [0,T-1]$), based on the learned system parameters and the respective inputs (features).

We establish an experimental scenario involving complete input-output data. To this end, 43 non-overlapping segments have been extracted from the 97 available episodes, based on the following conditions: (i) they are at least 400 frames long, so that the predictive capability of the proposed framework can be evaluated on long temporal segments portraying frequent conflict intensity fluctuations and conflict escalation/resolution, and (ii) the face detection for each frame is successful and, hence, the facial landmark detection results for each frame are accurate (see Chrysos et al. 2015, for further explanation).

The resulting subset of clips has a mean and standard deviation of duration of 804 frames and 561 frames, respectively, and corresponds to 22 subjects. For each of the 43 video sequences, the first $P=60\%$ of the frames are used for training, while the remaining frames are used for testing. This choice establishes a *subjects-dependent* experimental setting. It is worth mentioning that the experimental setting is challenging given that the proposed framework learns temporal behavioral patterns related to conflict escalation/resolution from a single dyadic interaction with average duration of about 19 seconds. This is in contrast to relying on a large set of training instances containing multiple interactants exhibiting conflicting behavior in various contexts.

For the proposed model (12), the following combinations are examined for the *p* and *q* values corresponding to the Schatten *p*- and $\ell _q$-norm, respectively: $(p,\,q) \in \{(1,2), (1,1), (0.9, 0.9), (0.5,0.5), (0.1,0.1) \}$. The scalable Algorithm 2 is also used for this experiment, with the dimension of the column space of $\mathbf {Q}$ in (26) set to $K=10$. The convergence parameters $\epsilon _1$ and $\epsilon _2$ are set to $10^{-4}$ and $10^{-7}$, respectively. For each sequence, 150 values, logarithmically spaced in the interval $[10^{-3},1 ]$ are examined for the tuning of parameter $\lambda$ in Algorithms 1 and 2. Similarly, a suitable grid search is conducted to tune the parameters of the compared methods. For details on methods to which we compare, see Table 1.

For evaluation, the Pearson Correlation Coefficient (COR) is used, measured between the ground truth $\mathbf {y_t}$ (mean over the 10 annotations) and the predicted output ${\hat{\mathbf {y}}_t}$ on the test set frames ($t \in [T_{train},T-1]$) of each sequence. Motivated by recent works on predictive analysis of human behavior (Mavadati et al. 2013; Kaltwang et al. 2016), we choose to also report the Intra-Class Correlation Coefficient (ICC), which was first proposed in Shrout and Fleiss (1979) as a metric to assess rater reliability in behavioral measurements. Specifically, the coefficient ICC(3,1) is employed herein, that corresponds to the case “*Each target is rated by each of the same* *k* *judges, who are the only judges of interest*” (Shrout and Fleiss 1979). For each sequence and method, the ICC(3,1) (henceforth denoted by ICC) is calculated by considering the ‘method’ and the ‘mean annotator’ as the only ‘judges’ of interest and the conflict intensity values for the test set frames as ‘targets’ in the definition above. To obtain a ‘human’ baseline ICC result, i.e., a measure of ‘level of consistency amongst 10 humans in measuring conflict intensity’, we also compute the ICC amongst the 10 annotations for the test frames of each sequence. The average value of the inter-annotator ICC, denoted by ICC$_h$, over all 43 sequences, was found ICC$_h=0.740$. Finally, note that each method is separately optimized in terms of each metric.

*Results and Discussion* Results in terms of mean value of COR and ICC over all 43 sequences are reported in Table 3 for all methods examined. For details on methods to which we compare, see Table 1. The values of the resulting LTI system order and execution time (time: secs per frame $\times$ 100) for the respective best-performing structured rank minimization solution are also reported, again averaged over all sequences.^4^ As can be seen, the proposed methods outperform all methods that are compared to, in terms of both COR and ICC. The second-best-performing method in terms of both metrics is IHTLS, with all remaining methods yielding lower scores. Results obtained by the scalable Algorithm 2 (denoted by ours$^{sc}$) are on par with those yielded by Algorithm 1. As a matter of fact, the best overall performance in terms of both metrics is achieved by the scalable algorithm with $p=q=0.1$. Furthermore, the proposed methods (12) and (26) yield superior performance when the objective function is non-convex ($p,\,q < 1$), as compared to that obtained by convex instances of (12) and instances of (26) with convex objective function ($p,\,q =1$ and $p=1,\,q=2$). These results indicate that the dynamic model learned with the non-convex instances explain better the observed data thus providing a better estimate for the system order than that learned with the convex instances. This may be attributed to the relaxation gap entailed by replacing the rank and $\ell _0$-norm with the Schatten *p*- and $\ell _q$-norm, respectively, is tighter than that entailed by convex approximations. Also, it is interesting to observe that the choice $q=2$, which corresponds to a Frobenius-norm based fitting measure, consistently results in the lowest performance amongst the values examined for the $\ell _q$-norm. Presumably, this is due to the susceptibility of the corresponding fitting measures to gross, sparse noise (Huber 2011).

**Table 3:** Conflict intensity prediction results in terms of COR and ICC, averaged over all 43 sequences used from the CONFER dataset

Method	Order	Time	COR	ICC
HRM	12	0.08	0.630	0.748
SVD-free	3	0.02	0.005	0.492
SRPCA	14	1.12	0.491	0.721
IHTLS	6	7.77	0.724	0.775
SLRA	7	1.34	0.637	0.708
ours ($p=1$, $q=2$)	4	0.22	0.565	0.762
ours ($p=1$, $q=1$)	5	0.26	0.771	0.817
ours ($p=0.9$, $q=0.9$)	6	0.35	0.800	0.824
ours ($p=0.5$, $q=0.5$)	7	0.59	0.805	0.811
ours ($p=0.1$, $q=0.1$)	9	0.70	0.801	0.822
ours$^{sc}$ ($p=1$, $q=2$)	4	0.19	0.671	0.772
ours$^{sc}$ ($p=1$, $q=1$)	5	0.26	0.789	0.813
ours$^{sc}$ ($p=0.9$, $q=0.9$)	6	0.34	0.788	0.827
ours$^{sc}$ ($p=0.5$, $q=0.5$)	5	0.68	0.781	0.815
ours$^{sc}$ ($p=0.1$, $q=0.1$)	5	0.83	**0.806**	**0.833**

The bold values indicate the best performances in terms of each evaluation metric

Averaged values for the resulting system order and execution time (time: secs per frame $\times$ 100) are also shown for each (COR-optimized) structured rank minimization method. For details on methods to which we compare, see Table 1

Regarding run time efficiency, it is worth noting that the execution time accounted for by the best-performing variant of the proposed methods (ours$^{sc}$ with $p=q=0.1$) is close to a degree of magnitude smaller than that of the best-performing out of the compared methods (IHTLS). As expected, execution time increases as *p* and *q* values move closer to zero. Moreover, the high COR and ICC scores achieved by the proposed methods are accompanied by low values for the resulting system orders (e.g., $n \in [4,6]$ for ours$^{sc}$). This property is crucial for both the generalizability and execution time efficiency of the overall predictive framework.

Notably, IHTLS, HRM and the proposed methods lead to an average ICC which is higher than the mean inter-annotator ICC$_h$ of 0.740. This means that these methods, which were trained using the ‘mean annotator’ annotations, have learned the trend of the ‘mean annotator’ exceptionally well and were able to reproduce the trend accurately. This clearly demonstrates the suitability of these methods for modeling the human behavior analysis task at hand (i.e., conflict intensity prediction).

*Effect of the Training Set Size on Prediction Accuracy* The results reported in Table 3 correspond to using the first $P=60\%$ of each sequence’s frames for training (structured rank minimization and LTI system learning) and the remaining frames for predicting the respective conflict intensity values. To investigate how the choice of the portion of frames used for training affects the predictive capability of the structured rank minimization-based framework, we vary the training set percentage *P* in $\{30\%,40\%,50\%,60\%,70\%\}$ of the sequence length. The test set percentages vary also according to 100-*P*. The resulting training (test) set sizes, averaged over all 43 sequences, are 240, 322, 402, 483, 563 (559, 482, 401, 321, 241) frames, respectively. For this experiment, the proposed method with $p=q=0.1$ is examined along with the same five compared methods, while performance is evaluated in terms of the COR metric only. For details on methods to which we compare, see Table 1.

**Fig. 4:**
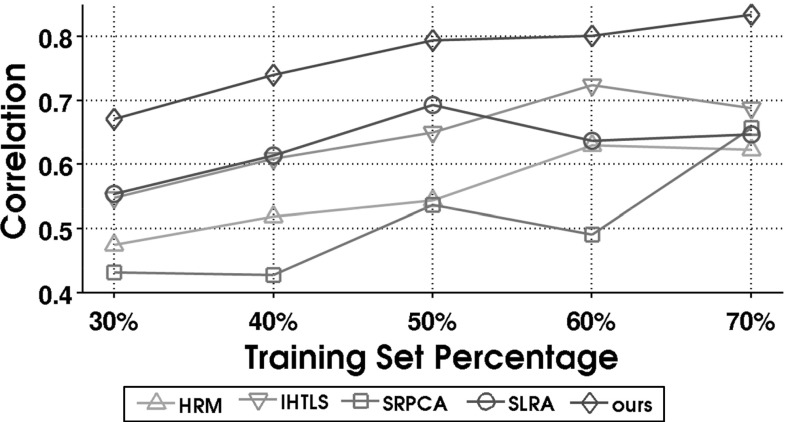
Average correlation (COR) values plotted as a function of the training set percentage, for the conflict intensity prediction experiment on the CONFER dataset with varying training size. For details on methods to which we compare, see Table 1. Results for the proposed method (12) were obtained by using Algorithm 1 with $p=q=0.1$

A graph that shows the COR values (averaged over all sequences) obtained for each percentage *P* by the various methods^5^ is illustrated in Fig. 4. The proposed method consistently outperforms the compared methods in all five scenarios. The second-best-performing method is SLRA and IHTLS for *P* in $$\{30\%, 40\%, 50\%\}$$ and *P* in $$\{60\%, 70\%\}$$, respectively. The superiority of the proposed method over the compared methods for this experiment is more evident in the cases where 30 or 40% of the frames are used for training; the discrepancy in performance achieved by the proposed method and SLRA reaches 0.117 and 0.126 in absolute COR terms, respectively. Overall, in most of the cases, a higher COR value is achieved by all methods when more data are used for training. For our method, the obtained COR values increase strictly monotonically with *P*, reaching $$ COR =0.834$$ at $$P=70\%$$.

**Fig. 5:**
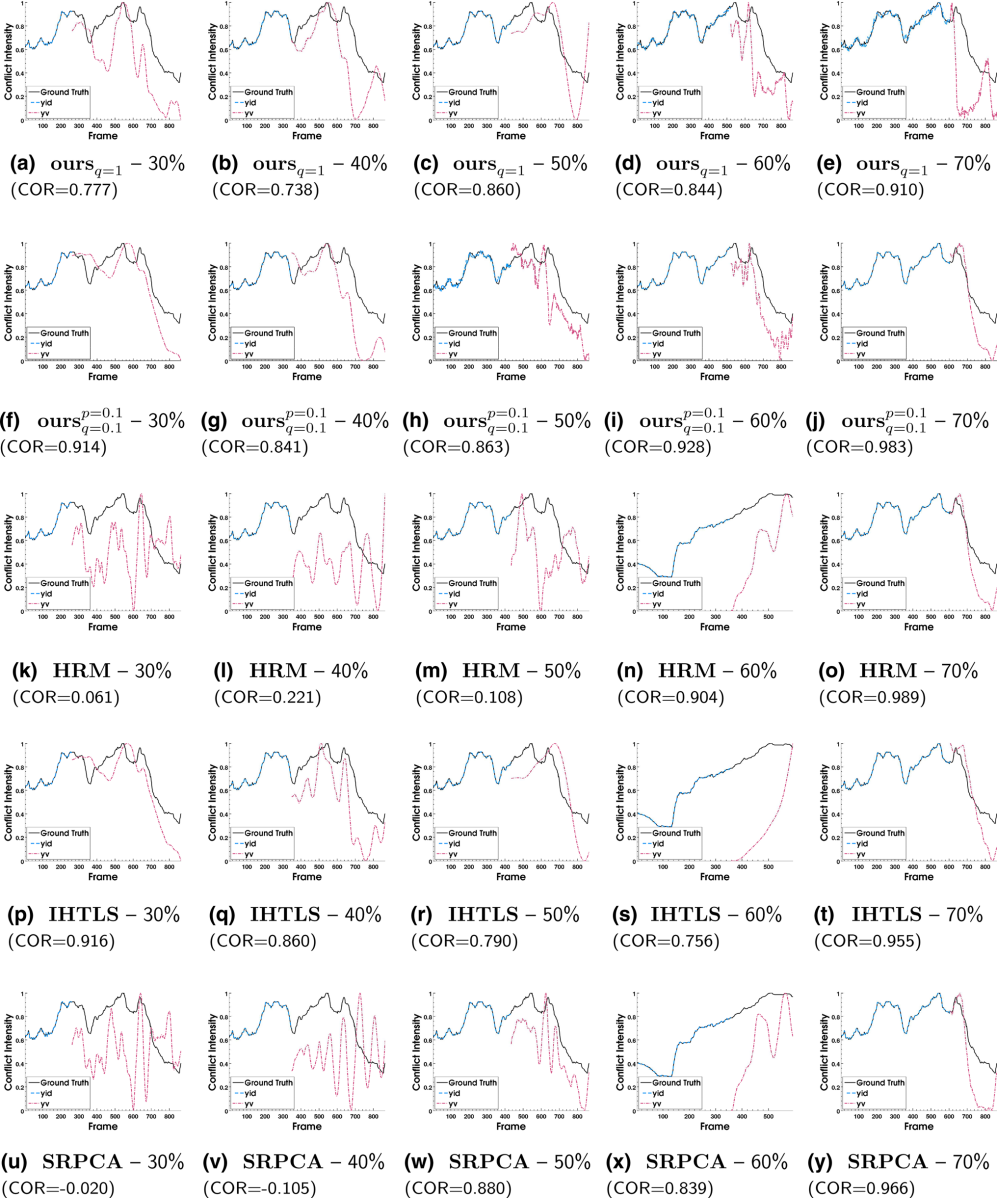
(Better viewed in *color*). Conflict intensity prediction results for a single sequence of the CONFER dataset, as produced by the proposed method ($(p,\,q) \in \{(1,1),(0.1,0.1)\}$), HRM, IHTLS and SRPCA for different portions of frames used for training (reported as percentages in the sub-captions along with the respective COR). For details on methods to which we compare, see Table 1. In each graph, the curve designated by ‘yid’ (‘yv’) corresponds to the training (test) predictions, while the third, *solid-line curve* corresponds to the ground truth annotations (mean over 10 ratings). The test set predictions have been normalized to the range [0,1] for better visualization (Color figure online)

In Fig. 5, conflict intensity predictions, as obtained by the proposed method ($(p,\,q) \in \{(1,1),(0.1,0.1)\}$), HRM, IHTLS and SRPCA for a sequence of the CONFER dataset, are illustrated along with the ground truth annotations as line plots for the various training set percentages examined. The COR values obtained are also shown in the respective sub-captions. As can be seen, the sequence in question establishes a challenging scenario, since it involves instances of both conflict escalation and resolution, either short- or long-term. One can easily notice that for all scenarios the trends of conflict intensity along the test frames are accurately predicted by the non-convex instance of the proposed method ($p=q=0.1$), while the convex model instance ($p=q=1$) yields smaller COR values in all five cases examined. The former achieves a COR value as high as 0.914 (Fig. 5f) for a total of 604 test frames when trained on just the first 30% of the sequence (260 frames). In the same scenario, IHTLS performs similarly, while other methods such as HRM and SRPCA yield COR values that lie just above or below zero, respectively. The various compared methods exhibit different patterns in performance as the amount of video frames used for training increases. For instance, IHTLS outperforms the other methods when less training data are used (30 and 40%), while SRPCA and HRM show a dramatic increase in performance at the point where 50 and 60% of the video frames are employed for training, respectively. The effectiveness of IHTLS in the scenarios involving less training data for the sequence in question is as expected. IHTLS is more likely to find a local approximation for the ‘low-complexity’ temporal dynamics of the first portion of the sequence that be low-rank and hence a simpler, more generalizable system than the convex, nuclear-norm based methods SRPCA and HRM, since the former searches for the desired rank iteratively starting from rank 1 (Dicle et al. 2013). Finally, as expected, the highest COR values obtained overall correspond to the highest training percentage of 70% and are similar across all methods.

**Fig. 6:**
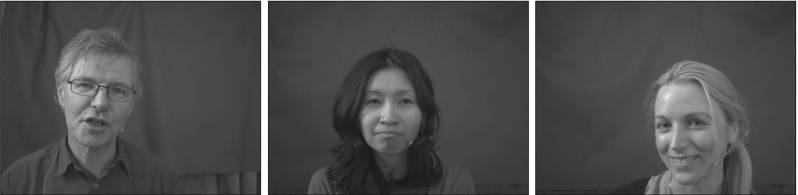
Example images from the SEMAINE database portraying three subjects from Session 46 (*left*), 82 (*middle*), and 94 (*right*)

### Valence and Arousal Prediction

In this section, the efficiency of the proposed dynamic behavior analysis framework is validated on the problem of *continuous prediction of valence and arousal* based on *visual features only*. Motivated by advances in psychology and cognitive neuroscience (Russell 1980; Lane and Nadel 2002), focus of affective computing research has recently shifted towards *continuous-time* analysis of affect phenomena, represented in the *dimensional* space (e.g., valence, arousal, power, anticipation) rather than in terms of universal basic emotions (e.g., happiness, surprise)  (Gunes and Schuller 2013; Gunes et al. 2011). *Valence* (how positive or negative the affect is) and *Arousal* (how excited or apathetic the affect is) are latent dimensions used to measure emotional experience, and are considered to encapsulate most of the affect variance (Lane and Nadel 2002).

Most of the existing automated approaches to Valence–Arousal (V–A) analysis have been limited to the use of audio cues only or have compromised to solving a two-class or four-class classification problem, i.e., binary classification with respect to each dimension or classification into the quadrants of the 2D V–A space (Gunes and Schuller 2013). Although the relation of affective dimensions (mostly arousal) to certain acoustic features has been better documented as compared to visual cues, yet there has been evidence that also visual signals (e.g., facial expressions, head shakes, nods) are informative of the V–A dimensions (Cowie et al. 2010; Pantic and Bartlett 2007). Such findings have motivated the exploitation of visual features, such as facial expression cues and shoulder movements, in either isolation or combination with audio features, for dimensional affect analysis. Representative examples of this line of research are the works of Gunes et al. (2011), Nicolaou et al. (2012) and Kaltwang et al. (2016).

In this paper, we address continuous prediction of valence and arousal using visual features only. Motivated by evidence suggesting that valence and arousal exhibit high correlation (Pantic and Bartlett 2007), we treat them in a joint framework, that is, as outputs generated by the same LTI system.

*Data* The SEMAINE database (McKeown et al. 2012), which contains audio-visual recordings of emotionally colored conversations between a human and an operator, is employed. The operator plays the role of an avatar and, depending on the choice of the latter, acts assuming one of 4 distinct personalities (happy, gloomy, angry or pragmatic). Since the goal of the operator is to elicit emotional reactions by the user, naturalistic dyadic conversations are developed, which are suitable for spontaneous affect analysis. Each video has been recorded at 50 frames per second, and has been annotated frame by frame by six raters in terms of real-valued valence and arousal ranging from −1 to 1. A subset of SEMAINE, containing 40 sequences that are at least 3000 frames ($\sim$1 min) long from a total of 10 subjects, is used. For each sequence, the mean values of valence and arousal annotations over the six ratings are utilized as ground truth. Three sample video frames corresponding to three different users from the SEMAINE database are depicted in Fig. 6.

**Table 4:** Valence (Val.) and Arousal (Ar.) prediction results in terms of COR and ICC, averaged over all 40 sequences used from the SEMAINE dataset

Method	Order		Time Val.	COR		ICC	
	Val.	Ar.		Val.	Ar.	Val.	Ar.
HRM	19	17	1.49	0.812	0.794	0.805	0.801
SVD-free	2	3	0.46	$-$0.024	0.001	0.504	0.412
SRPCA	16	21	5.95	0.771	0.743	0.774	0.765
IHTLS	10	9	121.14	0.727	0.739	0.739	0.734
SLRA	14	15	4.46	0.737	0.728	0.830	0.823
ours ($p=1$, $q=2$)	5	6	3.80	0.834	0.818	0.823	0.819
ours ($p=1$, $q=1$)	8	7	4.56	0.844	0.838	0.835	**0.835**
ours ($p=0.9$, $q=0.9$)	8	8	6.32	0.851	0.842	0.828	0.824
ours ($p=0.5$, $q=0.5$)	9	9	9.43	0.857	**0.871**	0.821	0.830
ours ($p=0.1$, $q=0.1$)	13	13	12.27	**0**.**866**	0.869	**0.837**	0.824

The bold values indicate the best performances in terms of each evaluation metric

Averaged values for the resulting system order (Val. and Ar.), and execution time (time: secs per frame $\times 100$) (Val.) are also shown for each (COR-optimized) structured rank minimization method. For details on methods to which we compare, see Table 1

*Features and Experimental Protocol* The Active Appearance Model-based tracker (Orozco et al. 2013), which performs simultaneous tracking of 3D head pose, lips, eyebrows, eyelids and irises in videos, is employed to extract facial features. For each frame, 113 2D characteristic facial landmarks are obtained. To ensure that only expression-related information is retained in the feature representation, we use the tracker’s estimates of 3D head pose values to remove pose angles. Scale and translation effects are subsequently removed from the 226 coordinates of the pose-normalized points, according to the procedure described for the experiment in Sect. 6.2. Finally, dimensionality reduction is performed by means of PCA. Again, 98% of the total energy is retained resulting to a 12-dimensional feature vector.

For each of the 40 sequences, the framework described in Sect. 5.1 is employed for continuous valence and arousal prediction. Only the first 3000 frames are considered for each sequence. The experimental protocol is similar to that established for the conflict intensity prediction experiment. The first 2000 frames of each sequence are used for training, while the remaining 1000 frames ($\sim$20 s) are used for V–A prediction. For this experiment, the visual feature vectors are used as inputs and the V–A values are used as outputs. Predictive performance for both valence and arousal is assessed again by means of both COR and ICC. To facilitate the evaluation and discussion with respect to each of the affect dimensions, we choose to optimize each method separately for each dimension and performance metric. For the proposed method, only Algorithm 1 is examined in this experiment. For details on methods to which we compare, see Table 1.

The mean value over all 40 sequences of the inter-annotator ICCh, calculated amongst the six available ratings, was found to be ICC$^{\text {V}}_h=0.778$ for valence and ICC$^{\text {A}}_h=0.893$ for arousal, respectively. The higher inter-annotator reliability for arousal is expected in the case of the SEMAINE data due to the three interlinked facts: (i) the majority of SEMAINE annotated data relate to high aroused emotions, (ii) the annotators were presented with audio-visual recordings to be annotated, and (iii) the arousal is better recognized when audio modality is available (Scherer et al. 2010; Bänziger and Scherer 2010).

*Results and Discussion* Valence and arousal prediction results, in terms of mean value of COR and ICC over all 40 SEMAINE sequences, are reported in Table 4 for all methods examined. For details on methods to which we compare, see Table 1. Mean values for the resulting system order and execution time (time: secs per frame $\times$100) are also reported.^6^ As can be seen, the best performance, in terms of both metrics, is obtained by the proposed method, for both valence and arousal prediction. The second-best-performing method in terms of COR (ICC) is HRM (SLRA) for both affect dimensions. Overall, valence and arousal are predicted with similar accuracies by almost all the methods. Again, the non-convex instances of the proposed method ($p,\,q < 1$) account for significant performance boost over convex model instances ($p,q =1$ and $p=1,q=2$), yet accompanied by an increase in model complexity and execution time. Still, in most of the cases the proposed method results in systems of lower-complexity, as compared to those accounted for by the remaining methods. Regarding execution time, the various methods achieve comparable performances, with the exception of IHTLS that is much slower for this experiment, probably due to the increased dimensions of the data Hankel matrices.

Finally, it is worth noting that the inter-annotator ICC$^{\text {V}}_h$ for valence is exceeded by HRM, SLRA and our method, whereas no method furnishes an ICC value greater than ICC$^{\text {A}}_h$ for arousal. This result is exactly as expected. Namely, as explained above, in the case of the utilized SEMAINE data, human annotators were presented with audio-visual (rather than visual-only) recordings when they were conducting the annotation. The presence of audio data does not affect the human performance in recognition of valence, but it does affect the recognition of arousal – arousal is better recognized when audio cues are available to humans to rely on (Bänziger and Scherer 2010). Hence, while automated methods like HRM and our methods are highly suitable for modeling human behavior analysis tasks at hand (i.e., valence intensity prediction), they could not learn the trends of the ‘mean annotator’ well enough for the case of arousal intensity prediction, because these were relying on audio data unavailable to the tested automated methods.

### Tracklet Matching

In this section, the efficiency of the proposed method is evaluated on the task of *tracklet matching*. The goal is to identify targets in the visual stream across occlusions from a set of given detections.

*Data* Experiments are conducted on the recently published Similar MultiObject Tracking (SMOT) dataset (Dicle et al. 2013), which consists of 8 videos^7^ showing multiple targets with identical or very similar appearance. For each video, the provided hand-labeled detections for the targets appearing in each frame are employed. Overall, the task is challenging due to the presence of multiple targets, long trajectories, object occlusions and crossings, missing data and camera motion.

*Features and Experimental Protocol* We follow the tracklet matching framework proposed in Dicle et al. (2013), which is based on a Generalized Linear Assignment (GLA) Problem. Thus, given *N* tracklets (trajectories of system outputs) $\{\mathbf {Y}^{(1)},\mathbf {Y}^{(2)},\ldots ,\mathbf {Y}^{(N)}\}$, GLA solves

33 $$\begin{aligned} \begin{aligned}&\max _{\mathbf {K}}{\sum _{i=1}^N \sum _{j=1}^N p_{ij}k_{ij}} \\&\quad \text{ s.t. } \sum _{i=1}^N k_{ij} \le 1; \, \sum _{j=1}^N k_{ij} \le 1; \, k_{ij} \in \{0,1\}, \end{aligned} \end{aligned}$$

where ${\mathbf {K}}$ is an adjacency matrix, with $k_{ij}=1$ denoting that $\mathbf {Y}^{(i)}$ is the predecessor of $\mathbf {Y}^{(j)}$, and ${\mathbf {P}}$ is a similarity matrix given by34with $\mathbf {Y}^{(ij)}=[\mathbf {Y}^{(i)} \; {\bar{\mathbf {y}}}_i^j \; \mathbf {Y}^{(j)}]$ being the joint tracklet of detections, padded with zeros at the entries of the tracklet ${\bar{\mathbf {Y}}}_i^j$ of missing data. Hence, the critical point of the aforementioned algorithm is the solution of the low-rank Hankel *matrix completion* problem $\min _{{\bar{\mathbf {y}}}_i^j}{\text {rank}(\mathcal {H}(\mathbf {Y}^{(ij)}))}$ in (34). This is solved according to the framework described in Sect. 5.2, in which the underlying LTI system is assumed to be autonomous and the data Hankel matrices are composed of the respective outputs (2D tracking point coordinates).

Two experimental scenarios are considered, similarly to (Dicle et al. 2013). In the first experiment, *false positives* are increased by injecting uniformly distributed false detections with percentage varying as $[0\%,10\%, \ldots , 50\% ]$. In the second scenario, *false negatives* are increased by removing, again uniformly, true detections with percentage varying as $[0\%,6\%, \ldots , 30\%]$. For each scenario, the experiment is repeated 10 times for each noise level, and the average performance over the 60 runs is reported. The same five methods used for comparison in the previous experiments are examined. For details on methods to which we compare, see Table 1. For the proposed method, Algorithm 1 is used, with the weight matrix $\mathbf {W}$ in (12) formed by setting its entries corresponding to the ‘missing’ tracklet ${\dot{\mathbf {Y}}}_i^j$ to zeros and all remaining entries to ones. Various values are examined for the parameters, that is, $(p,q) \in \{(1,2), (0.5, 2), (0.1,2)\}$ and $\lambda \in \{10^{-6}, 5 \cdot 10^{-6}, 10^{-5}, \ldots , 10^3 \}$, for each video and noise level. The convergence parameters $\epsilon _1$ and $\epsilon _2$ in Algorithm 1 are set to $10^{-7}$. For all methods examined, a Frobenius-norm based fitting measure is adopted ($q=2$ for the proposed method). This experimental choice was motivated by preliminary experiments, in which it was observed that the use of sparsity promoting norms for approximation error resulted in trivial solutions when a large amount of missing data was involved.

**Table 5:** Tracklet matching results, in terms of MOTA (Eq. (35)), on the SMOT dataset for each experimental scenario

Method	False positives		False negatives	
	Time	MOTA	Time	MOTA
HRM	0.202	0.9749	0.419	0.8687
SVD-free	0.033	0.9602	0.023	0.8422
SRPCA	0.104	0.9734	0.200	**0.8812**
IHTLS	0.174	**0.9799**	0.334	0.8712
SLRA	0.051	0.9646	0.230	0.7731
ours ($p=1$, $q=2$)	0.113	0.9733	0.249	0.8591
ours ($p=0.5$, $q=2$)	0.169	0.9745	0.277	0.8826
ours ($p=0.1$, $q=2$)	0.211	**0.9779**	0.311	**0.8880**

The bold values indicate the best performances in terms of each evaluation metric

For each noise type, the results are averaged over 6 noise levels, with each of the latter examined 10 times. Average execution time (time: secs per frame) accounted for by each structured rank minimization method is also shown. For details on methods to which we compare, see Table 1

**Fig. 7:**
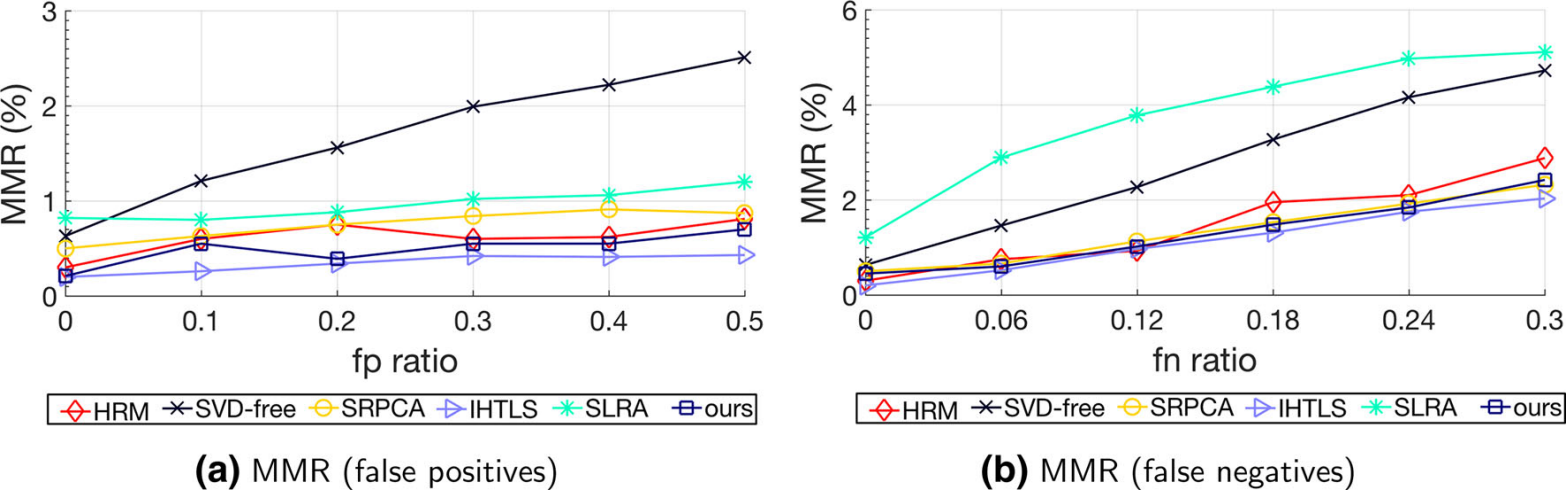
Tracklet matching results, as obtained by the proposed method ($p=0.1,q=2$) and the various compared methods, in terms of *MissMatch Ratio* $MMR = \frac{\sum _t{(mm_t)}}{\sum _t{g_t}}$ plotted as a function of noise level for the **a** false positives and **b** false negatives scenario, respectively

**Fig. 8:**
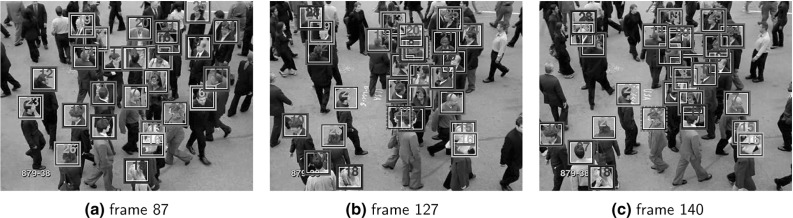
(Better viewed in *color*). Tracklet matching results, as produced by the proposed method (Algorithm 1 with $p=0.1$, $q=2$), illustrated on three frames of the *crowd* sequence from the SMOT dataset. The estimated trajectory index corresponding to each detection is shown inside a *bounding box*. *Solid line boxes* indicate given detections, while *dashed line boxes* indicate detections estimated by our method (Color figure online)

For evaluation, the MOTA measure (Bernardin and Stiefelhagen 2008) is used, which is given by

35 $$\begin{aligned} \mathrm{MOTA} = 1 - \frac{\sum _t{(fn_t+fp_t+mm_t)}}{\sum _t{g_t}} \, , \end{aligned}$$

where $fn_t$, $fp_t$, $mm_t$ and $g_t$ denote the false positives, false negatives, mismatches and ground truth detections for frame *t*, respectively.

*Results and Discussion* Tracklet matching results in terms of the MOTA measure—averaged over all 8 videos, noise levels and experiment runs—are reported for each scenario in Table 5. For details on methods to which we compare, see Table 1. Run time performance (time: secs per frame) of each respective algorithm, averaged similarly, is also reported. Overall, performance varies less amongst different methods for the false positives case, as compared to the false negatives case. This can be partially ascribed to the former case corresponding to a less demanding task of tracklet matching, since it involves a smaller amount of missing data. The proposed method performs similarly to IHTLS in terms of MOTA for both experimental scenarios, with the difference in performance for all 8 videos calculated as not statistically significant according to a paired *t*-test at significance level $\alpha =0.05$. All remaining methods achieve lower scores. The computational efficiency of the proposed method ($p=0.1,q=2$) is comparable to that accounted for by the best-performing amongst the compared methods, for both scenarios. Similarly to the previous experiments, the convex instance of our method ($p=1,q=2$) corresponds to a smaller execution time than that of the non-convex instances, albeit to a poorer performance.

Results in terms of *MissMatch Ratio* $MMR = \frac{\sum _t{(mm_t)}}{\sum _t{g_t}}$ plotted as a function of noise level, as obtained by the proposed method ($p=0.1,q=2$) and the various compared methods, are shown separately for the false positives and false negatives scenario in Fig. 7. By comparatively inspecting the two graphs, it is evident that more mismatches consistently occur in the false negatives scenario for all methods, which is exactly as expected. Also, MMR values vary slightly across noise levels in the false positives scenario for most methods, while in the most demanding false negatives scenario mismatches increase at a higher rate with the noise level. The best-performing methods for both cases are IHTLS and the proposed method, with the difference in MMR values being statistically insignificant according to a paired *t*-test at significance level $\alpha =0.05$ for all noise levels in both cases. On the other hand, the poorest performance for both cases is accounted for by the SVD-free and SLRA methods.

Tracklet matching results accounted for by the proposed method ($p=0.1$, $q=2$), shown as bounding boxes containing the estimated trajectory indices for the corresponding detections, are depicted on three characteristic frames of the *crowd* sequence from the SMOT dataset. The bounding boxes drawn with dashed lines correspond to detections estimated by the proposed method. One can observe that tracklets have been merged accurately in this challenging scenario that involves a heavily occluded surveillance scene. It is also worth noting that trajectory 22 (shown in red box) has been accurately ‘completed’ for frames 127 and 140 (Fig. 8b and 8c, resp.), despite the intense occlusion occurring at frame 127.

## Conclusions

A framework for dynamic behavior analysis in real-world conditions was developed in this paper. Specifically, the presented framework essentially employs a novel structured rank minimization method to learn a low-complexity system from time-varying data, in the presence of gross sparse noise and possibly missing data. By resorting to the ADMM, an efficient algorithm for the proposed structured rank minimization model along with a scalable version have been developed. Regarding applications, focus was placed on vision-based conflict intensity prediction, valence and arousal prediction, and tracklet matching. Extensive experiments on real-world data drawn from these application domains demonstrate the robustness and the effectiveness of the proposed framework.

## Footnotes

1 Note that for all experiments presented in this paper, the system order is defined as the rank of the estimated low-rank Hankel matrix, which is calculated as the number of singular values that are larger than 0.5% of the spectral norm, following Fazel et al. (2013).

2 The Dual AGP algorithm in Fazel et al. (2013) is used.

3
http://www.megatv.com/anatropi/.

4 The order and time values reported correspond to the COR-optimized methods.

5 COR values obtained by the SVD-free method are omitted from this discussion, as they were much lower compared to the other methods.

6 The order and time values reported correspond to the COR-optimized methods.

7 (1) *slalom* (three skiers), (2) *juggling* (3-ball juggling scene), (3) *acrobats*, (4) *seagulls*, (5) *TUD-Campus* (pedestrians), (6) *TUD-Crossing* (pedestrians), (7) *crowd* (from the crowd UCF dataset), (8) *balls* (bouncing identical ping pong balls).
